# Peripheral cytokines as candidate biomarkers for recurrent pregnancy loss

**DOI:** 10.1530/RAF-25-0152

**Published:** 2026-06-19

**Authors:** Danai Bagkou Dimakou, Jennifer Tamblyn, David Lissauer, Alex Richter

**Affiliations:** ^1^ University of Birmingham, Clinical Immunology Services, School of Infection, Inflammation and Immunology, Birmingham, UK; ^2^ Tommy’s National Centre for Miscarriage Research, Birmingham, UK; ^3^ University of Birmingham, School of Medical Sciences, Birmingham, UK; ^4^ Leeds NHS Teaching Hospital Trust, Leeds, UK; ^5^ University of Liverpool, Institute of Life Course and Medical Science, Liverpool, UK

**Keywords:** cytokines, pregnancy loss, RPL, biomarkers, inflammation

## Abstract

**Graphical Abstract:**

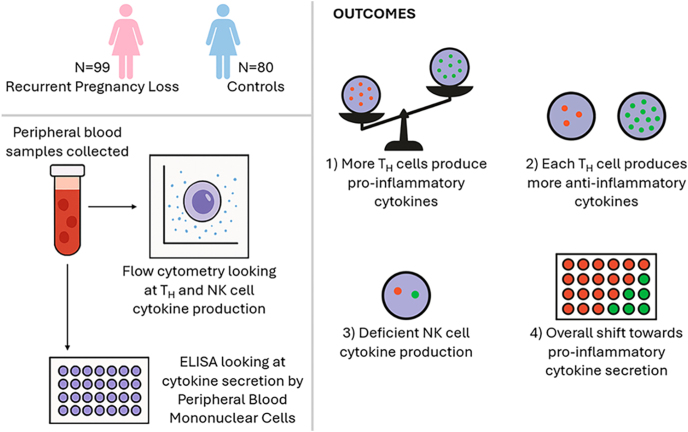

**Abstract:**

A current working hypothesis in recurrent pregnancy loss (RPL) suggests immune dysregulation, underlain by a pro-inflammatory cytokine shift in T_H_ cells. NK cells, which are primary immune effectors throughout pregnancy, also orchestrate immune activity via cytokine secretion, which appears aberrant in women with RPL. Experimental findings remain contentious, hindering clinical adoption of cytokine testing, despite increasing pressure from clinicians and couples affected. This study aims to identify peripheral cytokine biomarkers that could be utilized for the stratification of women with RPL displaying underlying immune dysregulation. Peripheral blood samples from non-pregnant women with RPL (*n* = 99) and controls (*n* = 80) were assessed for T_H_ and NK cell cytokine production via flow cytometry. Cytokine secretion by peripheral blood mononuclear cells was examined via ELISA. Elevated pro-/anti-inflammatory cytokine-producing T_H_ cell ratios were detected in RPL (*P* < 0.0001). This was, however, accompanied by reduced pro-/anti-inflammatory cytokine expression within individual cells (IL-2/IL-10: *P* < 0.0001, IFN-γ/IL-10: *P* < 0.0001, TNF-α/IL-10: *P* = 0.0136, IL-17A/IL-10: *P* < 0.0001). NK cell cytokine production appeared deficient overall in women with RPL (TNF-α^+^ NKs: *P* < 0.0001, IL-10^+^ NKs: *P* = 0.0006). Interestingly, a pro-inflammatory shift was evident when cytokine secretion was investigated (TNF-α/IL-5: *P* = 0.0120, IL-2/IL-13: *P* = 0.0105, IFN-γ/IL-13: *P* = 0.0040, TNF-α/IL-13: *P* < 0.0001). A pro-inflammatory shift in cytokine secretion was identified in women with RPL, indicating systemic cytokine dysregulation. Peripheral cytokine levels emerged as discriminatory and, thus, upon validation, may present valuable biomarkers in the identification of immune-associated RPL. Further research on the functional effects of cytokine imbalance *in utero* is required to strengthen the pathogenic relevance of these findings.

**Lay summary:**

Pregnancy losses (miscarriages) that happen repeatedly may be linked to an overactive immune system. We studied proteins that are used as signals to/from immune cells, called ‘cytokines’, in blood from women with repeated miscarriages and women without this history. We measured the production of these molecules by two immune cell types that are key in pregnancy: T helper cells and natural killer cells and their total levels in blood. In women with miscarriages, more T helper cells produced immune-activating over immune-suppressing cytokines. Natural killer cells made fewer cytokines overall. When we looked at their total levels in the blood, more immune-activating cytokines were seen. Our findings point to a change towards more immune activation in women with multiple miscarriages. After examining this further, cytokines could be used as a test to identify women who are more likely to benefit from treatments that help balance the immune system.

## Introduction

Each minute, ∼44 women worldwide experience pregnancy loss, resulting in 23,000,000 miscarriages/year (Quenby *et al.* 2021). Recurrent pregnancy loss (RPL), defined as 2 or more losses, affects 3% of the population, being linked to significant psychological distress, additional obstetric complications, and long-term health issues (ESHRE Guideline Group on RPL *et al.* 2018, Quenby *et al.* 2021).

Cytokines orchestrate maternal immune adaptation to pregnancy through a delicate balance between pro- and anti-inflammatory signals. Precise modulation appears essential for processes such as implantation, trophoblast invasion, and placental development. Disruptions in cytokine equilibrium can lead to an environment that is either excessively pro-inflammatory, potentially leading to out-of-phase implantation, inadequate embryo selection, and impaired placentation, or insufficiently immunoprotective, compromising tolerance (Ewington *et al.* 2019, Ticconi *et al.* 2019). Accumulating evidence suggests that cytokine imbalance, particularly shifts in T and NK cell-derived cytokines, may play a crucial role in RPL pathogenesis by altering the maternal–fetal immune interface and disrupting pregnancy maintenance (Bagkou Dimakou *et al.* 2022*a*).

CD4^+^ T_H_ cells play a central role in shaping adaptive immunity, being termed the most prolific cytokine producers. Their cytokine production is strongly linked to reproductive success, with increased pro-inflammatory IL-2/IFN-γ/TNF-α-producing T_H_1 and reduced regulatory IL-4/IL-5/IL-10/IL-13-secreting T_H_2 cells observed in RPL, termed ‘T_H_1/T_H_2 hypothesis’ (Calleja-Agius & Brincat 2008). The updated ‘type-1/type-2’ theory includes pro-inflammatory T_H_17 cells, secreting IL-17, IL-21, IL-22, and tolerogenic IL-10-producing Tregs (Moore *et al.* 2001, Roomandeh *et al.* 2018). A type-1 shift, characterized by increased T_H_1 and T_H_17 and reduced T_H_2 and Treg activity, has been widely observed in RPL, both in peripheral blood and *in utero* (Marzi *et al.* 1996, Wang *et al.* 2010, Ali *et al.* 2021).

Although systemic immunity is not reflective of *in utero* conditions, cytokine variation may be an inherent feature of certain RPL cases. Evidence supports such a hypothesis. For instance, T_H_17 cell/Treg cell, cytokine, and respective RORγt/FoxP3 transcription factor ratios appear elevated in both peripheral blood and decidua (Wang *et al.* 2010, 2013, Sereshki *et al.* 2014, Zhu *et al.* 2017). IL-10, IFN-γ, and TNF-α gene polymorphisms are also associated with RPL (Reid *et al.* 2001, Daher *et al.* 2003). Increased type-1 cytokines may promote cytotoxic anti-fetal activity, an effect counterbalanced by type-2 cytokine release (Carp 2004, Saini *et al.* 2011, Ali *et al.* 2021). IFN-γ and TNF-α impair implantation and embryonic development in murine models (Yockey & Iwasaki 2018, Ali *et al.* 2021), with IL-2 perpetuating their inflammatory effects. IL-17 acquires an important role, with elevated maternal levels linked to direct effects on fetal brain development (Fujitani *et al.* 2022, Kim 2023). IL-10 exhibits a protective anti-inflammatory role (Cheng & Sharma 2015).

Uterine NK cells are key pregnancy mediators, constituting up to 90% of lymphocytes in the maternal–fetal interface during early pregnancy. The local NK cell population consists primarily of CD56^bright^ cells that are less cytotoxic than CD56^dim^ ones but are more potent cytokine producers, indicating the importance of cytokine production during pregnancy establishment. These cytokines include TNF-α, IFN-γ, IL-10, IL-8, and GM-CSF, contributing to spiral artery remodelling, trophoblast growth, and placental development (Saito *et al.* 1993, Hanna *et al.* 2006, Lash *et al.* 2006*b*, Mor *et al.* 2011, Ticconi *et al.* 2019, Bagkou Dimakou *et al.* 2022*a*). In alignment with the type-1/type-2 hypothesis, an anti-inflammatory shift in NK cell cytokine production was observed in healthy pregnancy, characterized by reduced IFN-γ and enhanced IL-10 (Higuma-Myojo *et al.* 2005). In contrast, a persistent type-1 shift, marked by elevated pro-inflammatory NK cell cytokines, has been reported in the peripheral blood and decidua of women with RPL and those experiencing miscarriage (Higuma-Myojo *et al.* 2005, Fukui *et al.* 2008, Zhu *et al.* 2018, Liu *et al.* 2022). Notably, uterine NK cell-conditioned medium from first-trimester miscarriage samples was found to impair endometrial stromal cell decidualization, largely through TNF-α activity (Fonseca *et al.* 2020). Direct contact with extravillous trophoblasts, which invade the decidua during early pregnancy, suppresses NK cell cytokine secretion (Lash *et al.* 2011), indicating a potential fetal-mediated regulatory mechanism.

Overall, cytokine balance is instrumental to pregnancy success, with a significant pro-inflammatory skew in cytokine ratios reported in RPL, pre-pregnancy, and during early pregnancy (Raghupathy *et al.* 2000, Kwak-Kim *et al.* 2003, Marron *et al.* 2019, Kuroda *et al.* 2021). However, the idea of a universal anti-inflammatory shift is likely an oversimplification, as depending on its stage, pregnancy can be dominated by either pro- or anti-inflammatory processes. The type-1/type-2 hypothesis has not been consistently demonstrated, with type-2 dominance reported in the circulation and uterus of women with RPL (Bates *et al.* 2002, Fukui *et al.* 2017, Arora *et al.* 2020). T_H_1 cytokines are also essential for immunosurveillance during pregnancy and control trophoblast invasion and spiral artery remodelling (Ashkar *et al.* 2000, Carp 2004, Lash *et al.* 2006*a*, Otun *et al.* 2011).

A dynamic balance between cytokine responses appears critical. We hypothesize that a peripheral pro-inflammatory cytokine shift is present in women with pregnancy loss and could provide a valuable indicator of the degree and direction of aberrant immunity. This study aims to comprehensively analyse cytokine patterns in the peripheral blood of non-pregnant women and evaluate their diagnostic potential. To achieve this, we have utilized flow assays, which are used in certain private miscarriage clinics for the diagnosis of ‘immune-associated’ RPL, accompanied via ELISA that offers a more accurate insight into secreted cytokine amounts.

## Materials and methods

Non-pregnant women with RPL (*n* = 99), defined by the presence of ≥2 losses in their reproductive history, were recruited at Birmingham Women’s Hospital (16/WM/0423, IRAS:215646) and University Hospital Coventry & Warwickshire (17/WM/0050, IRAS:213740). Non-pregnant control women (*n* = 80) recruitment was performed at Birmingham Women’s Hospital (14/WM/1146, IRAS:155401). All participants were of reproductive age (≥18 years old and premenopausal) and recruited upon providing informed consent. Sample collection time was unstandardized due to pre-existing clinics’ schedule. Women using immunomodulatory agents and women with chronic or active systemic infections were excluded. No menstrual cycle standardization was performed, with participants recruited at different menstrual cycle phases. Due to the high number of participants recruited, our cohort is expected to reflect the general population. The clinic schedule allowed participants to rest for >30 min prior to sampling to control the influence of physical stress. Blood was collected, and peripheral blood mononuclear cells (PBMCs) were isolated using Vacutainer^®^CPT^TM^ tubes (BD Biosciences, USA). Demographic data were collected via Tommy’s Net (Khan *et al.* 2022).

Fresh PBMCs were cultured in complete RPMI, supplemented with 10% FBS and 1% penicillin/streptomycin, at a concentration of 10^6^ cells/mL. Cells were stimulated with 0.5 μg/mL ionomycin and 0.025 μg/mL phorbol 12-myristate 13-acetate (PMA) (Sigma-Aldrich, Germany) and incubated at 37°C, 5% CO_2_ for 30 min. Two cultures/samples were generated, for assessment of cytokine production by flow cytometry and cytokine secretion by enzyme-linked immunosorbent assay (ELISA). Unstimulated controls were also set up, allowing us to assess responsiveness to stimulation. Incubation was followed by the addition of 0.7 μL/mL GolgiStop^TM^ (BD Biosciences) to cultures used for flow cytometry. Cultures were incubated for 12 h at 37°C, 5% CO_2_ and were centrifuged at 500 *g* for 10 min. Pellets of GolgiStop^TM^-containing cultures were stained and examined by flow cytometry, while supernatants of non-GolgiStop^TM^-containing cultures were stored in −80°C for examination by ELISA.

Fresh unstimulated PBMCs were utilized for assessment of T cell population and subpopulation prevalence (Supplementary Table 1A, Supplementary Fig. 1 (see section on Supplementary materials given at the end of the article)). For FoxP3 staining, cells were permeabilized (eBioscience™ Foxp3/Transcription Factor Staining Buffer Set). Stimulated and unstimulated PBMCs were stained for T_H_ and NK cytokine production evaluation (Supplementary Tables 1B and 1C, Supplementary Figs 2 and 3). Per cell cytokine intensity was also examined. For intracellular cytokine staining cells were fixed and permeabilized (Fixation/Permeabilization Solution Kit with GolgiStop™, BD Biosciences). Fc block (BioLegend) was added to all tubes to limit unspecific staining. Data were obtained using the FACSCanto™ II cytometer and FACSCanto™ Software and processed on FlowJo^TM^ v10.6.2, with FlowAI 2.0 used for data clean-up.

Thawed supernatants from stimulated and unstimulated PBMC cultures were used for cytokine secretion assessment via the IL-2/IL-4/IL-5/IL-10/IL-13/IL-17A/IFN-γ/TNF-α/G-CSF/GM-CSF/IL-8 ProcartaPlex^TM^ Immunoassay (ThermoFisher Scientific, USA). The assay was run on Luminex™200™ and Bio-Plex Manager Software 6.1. Standards met included microsphere aggregates <30%, microsphere counts >50/sample, 70 ≤ standard recovery rate ≤ 130, and duplicate standard dilution coefficient of variation <20%.

Statistical analysis was performed on GraphPad Prism 6. Data were not log-transformed or otherwise edited. The distribution of each dataset was evaluated via the D’Agostino–Pearson omnibus normality test. In the case of unpaired data (controls vs RPL, controls vs idiopathic RPL, non-idiopathic vs idiopathic RPL, nulliparous vs parous controls, RPL vs parous controls), when both datasets compared were normally distributed, a parametric unpaired *t* test was used. When at least one of the datasets was non-normally distributed, the non-parametric Mann–Whitney test was utilized. For paired data (unstimulated vs stimulated), a parametric paired *t* test was used when both datasets were normally distributed and a non-parametric Wilcoxon matched-pairs signed rank test was performed when at least one dataset was non-normally distributed. When more than two unpaired datasets were compared (age and ethnic subgroups), Kruskal–Wallis and Dunn’s tests were used as all datasets were non-normally distributed. In all tests, an *α* = 0.05 was used.

A correction for multiple comparisons was not applied as the cytokines assessed were individually selected based on past publications indicating a significant involvement in pregnancy loss. Although the number of comparisons for each dataset was limited, the possibility of false discoveries cannot be excluded and further validation of markers of interest should be performed. Parameters that were found to be significantly different between women with RPL and controls were normalized by division against live birth probability, calculated using the Tommy’s live birth calculator (https://pavlev.shinyapps.io/lbcalculator/, based on age and pregnancy history). Normalized RPL and control data were unpaired and non-normally distributed, and thus, the Mann–Whitney test was used.

## Results

### Participants’ characteristics and cohort differences

Significant demographic differences, including increased age and gravidity, were seen between cohorts, alongside ethnic diversity, with an Asian population overrepresentation in the RPL group. Abstinence from alcohol was more prevalent in women with RPL. Blood samples were collected at a later time from control than RPL participants, although 95% confidence intervals indicate that most samples from both groups were collected in the afternoon, between ∼12:00 and ∼15:00 h (Table 1). Most women with RPL had 2 or 3 previous losses and 53/99 presented with the idiopathic form of this condition (Supplementary Fig. 4). No participants reported illicit drug use.

**Table 1:** Summary of participant characteristics. The age, gravidity, parity, body mass index (BMI), ethnicity, smoking habits, and average weekly alcohol intake of women in the RPL (*n* = 99) and control (*n* = 80) groups, as well as the time of sample collection (converted into decimal hours) for both groups are depicted, alongside the respective *P* values.

Characteristics	RPL	Control	*P* value
Age (years)*	33 (32.01, 34.23)	30 (30.08, 33.67)	0.0430
Gravidity*	4 (3.57, 4.41)	0 (0.81, 1.52)	<0.0001
Parity*	0 (0.41, 0.76)	0 (0.60, 1.16)	0.3253
BMI (kg/m^2^)*	25.00 (25.32, 27.77)	26.47 (25.75, 28.57)	0.5052
Ethnicity^†^			0.0007
Asian	21	5	
Black	2	8	
White	57	59	
Mixed	1	6	
Smoking status^‡^			0.5694
Non-smokers	91	72	
Smokers	6	7	
Alcohol intake (units/week)^†^			<0.0001
0	69	31	
1–7 (low)	27	29	
8–14 (moderate)	1	16	
>14 (high)	1	3	
Time of blood collection (h)*	12.25 (12.19, 13.21)	14.53 (13.98, 15.03)	<0.0001

* For non-normally distributed data, Mann–Whitney test used; median (95% confidence interval) displayed.

^†^ Chi-square test used; number of women displayed.

^‡^ Fisher’s exact test used; number of women displayed.

### Reduced T cell abundance with preserved subset distribution in RPL

**Figure 1:**
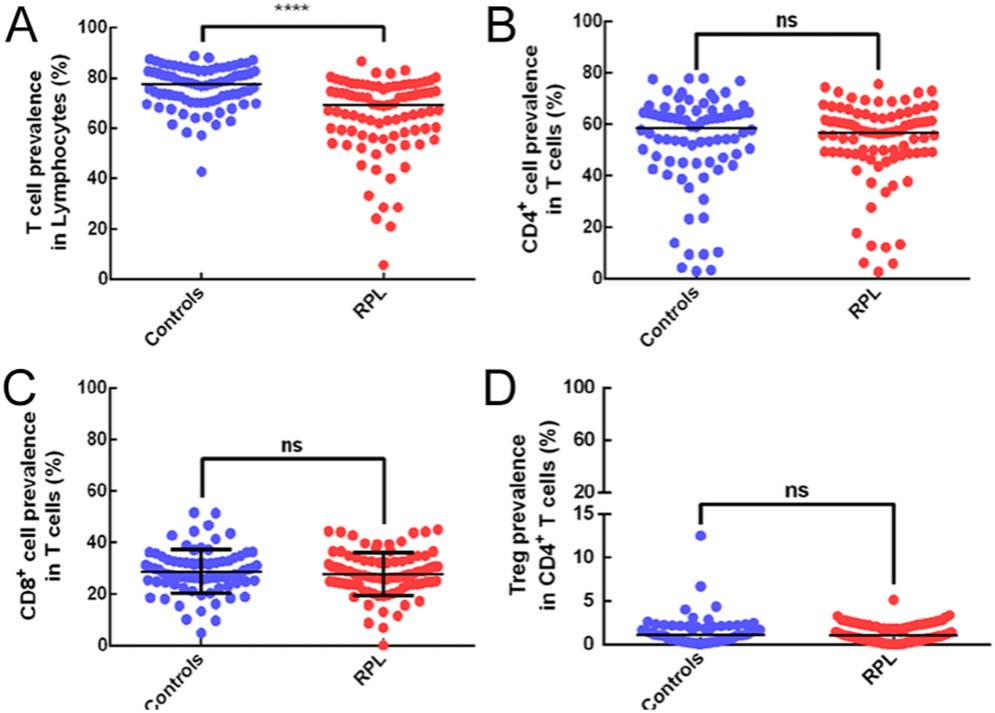
T cell population and subpopulation prevalence in control women and women with recurrent pregnancy loss (RPL). (A) CD3+ T cell prevalence in the peripheral blood lymphocyte population; (B) CD4+CD8− and (C) CD4−CD8+ subpopulation prevalence in T cells; and (D) FoxP3+CD25+CD127low/− regulatory T cell (Treg) prevalence in CD4+CD8− T cells in controls (*n* = 80) and women with RPL (*n* = 89) are depicted. Normally distributed datasets (C): unpaired *t* test performed; mean and standard deviation shown. Non-normally distributed datasets (A, B, D): Mann–Whitney test used; median displayed. ns, non-significant; ****P ≤ 0.0001.

Peripheral T cell prevalence was decreased in RPL (controls: 77.50% (73.70%, 80.50%), RPL: 69.20% (64.50%, 73.00%), *P* < 0.0001), accompanied by unaltered CD4^+^ (controls: 58.45% (53.70%, 62.60%), RPL: 56.70% (54.30%, 60.20%)), CD8^+^ (controls: 28.82% (8.57), RPL: 27.80% (8.42)), and Treg (controls: 1.09% (0.70%, 1.51%), RPL: 1.03% (0.72%, 1.44%)) subpopulation percentages (Fig. 1).

### Imbalance of T_H_ cell cytokine production in RPL

We next examined whether altered cytokine production by T_H_ cells could explain immune imbalance observed in women with RPL. In the absence of exogenous stimulation, TNF-α^+^ and IL-10^+^ T_H_ percentages appeared decreased in RPL (*P* < 0.0001). However, the intensity of TNF-α expression was augmented (*P* < 0.0001) and IL-10 expression intensity was unaltered (Supplementary Fig. 5C, 5D, 5H, 5I). Baseline IL-2, IFN-γ, and IL-17A production was unaltered (Supplementary Fig. 5A, 5B, 5E, 5 G, 5J).

**Figure 2:**
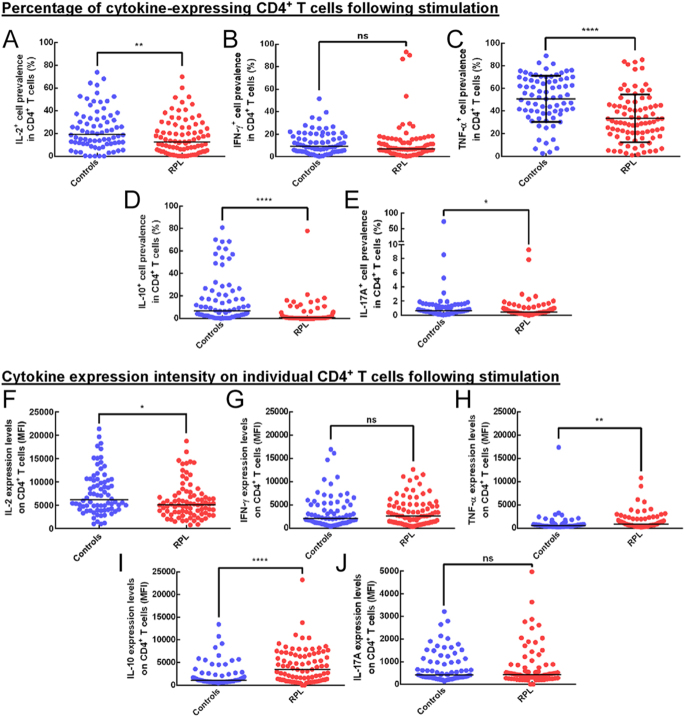
Cytokine production by stimulated T_H_ cells of women with recurrent pregnancy loss (RPL) and controls. (A) IL-2^+^, (B) IFN-γ^+^, (C) TNF-α^+^, (D) IL-10^+^, and (E) IL-17A^+^ CD4^+^ T_H_ cell percentages and expression intensity of (F) IL-2, (G) IFN-γ, (H) TNF-α, (I) IL-10, and (J) IL-17A on individual CD4+ T_H_ cells, represented by the median fluorescence intensity (MFI), following stimulation, in healthy controls (*n* = 76) and women with RPL (*n* = 82) are displayed. Normally distributed datasets (C): unpaired *t* test performed; mean and standard deviation depicted. Non-normally distributed datasets (A, B, D, E, F, G, H, I, J): Mann–Whitney test used; median illustrated. ns, non-significant; *P ≤ 0.05; **P ≤ 0.01; ****P ≤ 0.0001.

Upon stimulation, IL-2^+^ (*P* = 0.0084) and TNF-α^+^ (*P* < 0.0001) cell percentages were reduced in the RPL group (Fig. 2A and C). IL-2 expression levels were also decreased in RPL (*P* = 0.0121). TNF-α levels appeared elevated (*P* = 0.0016) (Fig. 2F and H). Although fewer cells expressed IL-10 (*P* < 0.0001), IL-10 expression levels were augmented in RPL (*P* < 0.0001) (Fig. 2D and I). IL-17A^+^ T_H_ cell percentage was lower (*P* = 0.0362), with the respective expression levels appearing unaltered (Fig. 2E and J). Similar to the unstimulated state, stimulated T_H_ cells’ IFN-γ production was not significantly different between cohorts (Fig. 2B and G).

**Figure 3:**
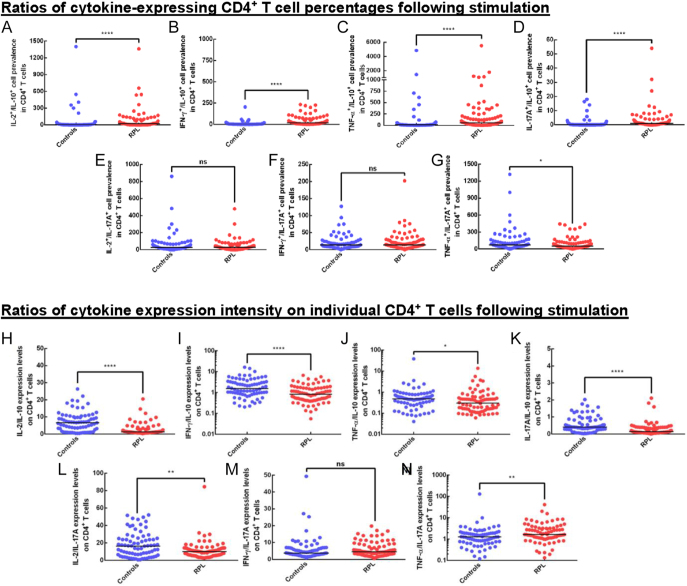
Ratios of cytokine production by stimulated T_H_ cells of women with recurrent pregnancy loss (RPL) and controls. (A) IL-2^+^/IL-10^+^, (B) IFN-γ^+^/IL-10^+^, (C) TNF-α^+^/IL-10^+^, (D) IL-17A^+^/IL-10^+^, (E) IL-2^+^/IL-17A^+^, (F) IFN-γ^+^/IL-17A^+^, and (G) TNF-α^+^/IL-17A^+^ CD4^+^ TH cell percentages and expression intensity of (H) IL-2/IL-10, (I) IFN-γ/IL-10, (J) TNF-α/IL-10, (K) IL-17A/IL-10, (L) IL-2/IL-17A, (M) IFN-γ/IL-17A, and (N) TNF-α/IL-17A on individual CD4+ T_H_ cells, represented by the median fluorescence intensity (MFI), following stimulation with phorbol 12-myristate 13-acetate and ionomycin, in healthy controls (*n* = 76) and women with RPL (*n* = 82) are illustrated. For these non-normally distributed datasets, the Mann–Whitney test was used and median is shown. ns, non-significant; *P ≤ 0.05; **P ≤ 0.01; ****P ≤ 0.0001.

Pro-/anti-inflammatory cytokine production ratios following T_H_ cell stimulation are often examined in RPL clinics. The ratios of pro-inflammatory IL-2-, IFN-γ-, TNF-α-, or IL-17A-expressing to anti-inflammatory IL-10^+^ T_H_ cells were increased in RPL (*P* < 0.0001) (Fig. 3A, B, C, D). The respective expression level ratios were reduced (IL-2/IL-10, IFN-γ/IL-10, and IL-17A/IL-10: *P* < 0.0001, TNF-α/IL-10: *P* = 0.0136) (Fig. 3H, I, J, K). T_H_1/T_H_17-associated ratios were also assessed. The TNF-α^+^/IL-17A^+^ T_H_ ratio was decreased in RPL (*P* = 0.0434), while the IL-2^+^/IL-17A^+^ and IFN-γ^+^/IL-17A^+^ T_H_ ratios were unaltered (Fig. 3E, F, G). Regarding expression levels, the TNF-α/IL-17A ratio was elevated (*P* = 0.0031), IL-2/IL-17A was reduced (*P* = 0.0087), and IFN-γ/IL-17A was unaltered in RPL (Fig. 3L, M, N). Thus, dysregulated cytokine production was observed in women with RPL, without however displaying an evident pro- or anti-inflammatory direction.

### Defective NK cell cytokine production in RPL

We assessed whether cytokine production by NK cells is also affected in RPL. Stimulation induced elevated IFN-γ and TNF-α production by NK cells in both the RPL and control groups (Supplementary Table 2). However, IL-10^+^ NK cell percentage was unaltered upon stimulation, while IL-10 expression intensity was only increased in control and not RPL cases (Supplementary Table 2).

In the absence of stimulation, TNF-α^+^ (*P* < 0.0001) and IL-10^+^ (*P* = 0.0468) NK cell percentages, but not their expression intensities, appeared reduced in RPL (Supplementary Fig. 6B, 6C, 6E, 6F). Unstimulated NK cells’ IFN-γ expression was unaltered (Supplementary Fig. 6A and 6D).

**Figure 4:**
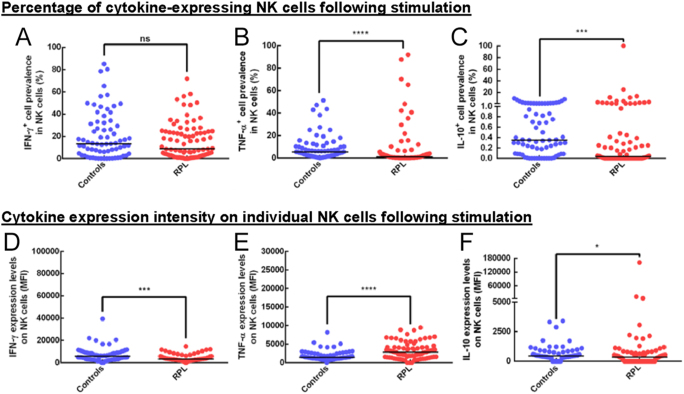
Cytokine production by stimulated NK cells of women with recurrent pregnancy loss (RPL) and controls. (A) IFN-γ^+^, (B) TNF-α^+^, and (C) IL-10^+^ NK cell percentages and expression intensity of (D) IFN-γ, (E) TNF-α, and (F) IL-10 on individual NK cells, represented by the median fluorescence intensity (MFI), following stimulation with phorbol 12-myristate 13-acetate and ionomycin, in healthy controls (*n* = 71) and women with RPL (*n* = 84) are displayed. For these non-normally distributed datasets, the Mann–Whitney test was used and median is shown. ns, non-significant; *P ≤ 0.05; ***P ≤ 0.001; ****P ≤ 0.0001.

Following stimulation, TNF-α^+^ (*P* < 0.0001) and IL-10^+^ (*P* = 0.0006), but not IFN-γ^+^ cell percentage was reduced in RPL (Fig. 4A, B, C). TNF-α (*P* < 0.0001) expression levels were elevated but IFN-γ (*P* = 0.0002) and IL-10 (*P* = 0.0452) levels were decreased (Fig. 4D, E, F).

**Figure 5:**
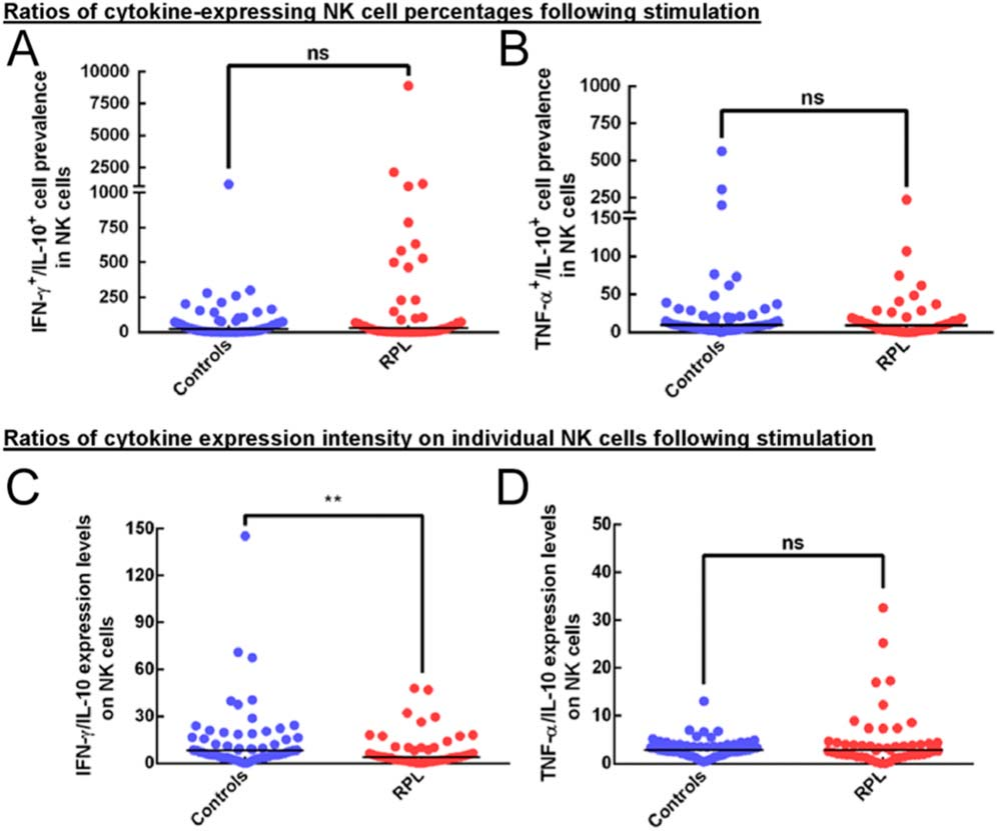
Ratios of cytokine production by stimulated NK cells of women with recurrent pregnancy loss (RPL) and controls. (A) IFN-γ^+^/IL-10+ and (B) TNF-α^+^/IL-10^+^ NK cell percentages and expression intensity of (C) IFN-γ/IL-10 and (D) TNF-α/IL-10 on individual NK cells, represented by the median fluorescence intensity (MFI), following stimulation with phorbol 12-myristate 13-acetate and ionomycin, in healthy controls (*n* = 71) and women with RPL (*n* = 84) are illustrated. For these non-normally distributed datasets, the Mann–Whitney test was used and median is shown. ns, non-significan; **P ≤ 0.01.

IFN-γ^+^/IL-10^+^ and TNF-α^+^/IL-10^+^ NK cell ratios appeared unaltered in RPL (Fig. 5A and B). IFN-γ/IL-10 expression intensity was reduced (*P* = 0.0061), while the TNF-α/IL-10 intensity was unaltered (Fig. 5C and D). In summary, no clear direction of the NK cell cytokine dysregulation could be identified.

### Pro-inflammatory shift in PBMC cytokine secretion in RPL

To determine whether cellular cytokine changes translated into an altered cytokine output, we examined secretion by total PBMCs. Although stimulation resulted in increased secretion of most cytokines in both study cohorts, IL-8 and TNF-α secretion was elevated upon stimulation in controls, but not RPL. G-CSF secretion reduced upon stimulation in women with RPL and controls. IL-10 levels decreased following stimulation, but only in the RPL group (Supplementary Table 2).

In the absence of stimulation, TNF-α (*P* = 0.0313), IL-17A (*P* < 0.0001), IL-8 (*P* = 0.0006), and GM-CSF (*P* = 0.0022) were increased and IL-2 (*P* = 0.0080) and IL-5 (*P* < 0.0001) levels were reduced in RPL (Supplementary Fig. 7A, 7C, 7D, 7G, 7I, 7K).

**Figure 6:**
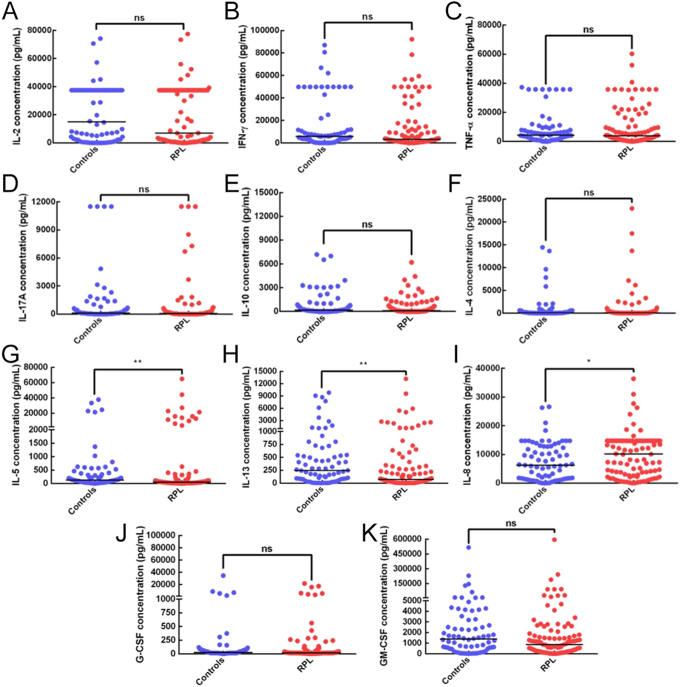
Protein concentration (pg/mL) in stimulated peripheral blood mononuclear cell (PBMC) cultures derived from women with recurrent pregnancy loss (RPL) and controls. The levels of (A) IL-2, (B) IFN-γ, (C) TNF-α, (D) IL-17A, (E) IL-10, (F) IL-4, (G) IL-5, (H) IL-13, (I) IL-8, (J) G-CSF, and (K) GM-CSF secretion by PBMCs isolated from healthy controls (*n* = 72) and women with RPL (*n* = 99), following stimulation with phorbol 12-myristate 13-acetate and ionomycin, are illustrated. For these non-normally distributed datasets, the Mann–Whitney test was used and median is displayed. ns, non-significant; *P ≤ 0.05; **P ≤ 0.01.

Upon stimulation, participants with RPL displayed reduced IL-5 (*P* = 0.0016) and IL-13 (*P* = 0.0030) and increased IL-8 (*P* = 0.0276) secretion (Fig. 6G, H, I).

**Figure 7:**
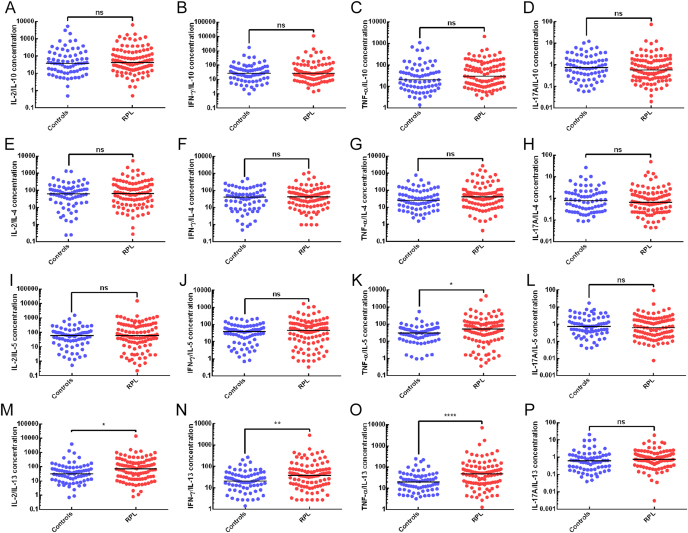
Protein concentration ratios in stimulated peripheral blood mononuclear cell (PBMC) cultures derived from women with recurrent pregnancy loss (RPL) and controls. The ratios of (A) IL-2/IL-10, (B) IFN-γ/IL-10, (C) TNF-α/IL-10, (D) IL-17A/IL-10, (E) IL-2/IL-4, (F) IFN-γ/IL-4, (G) TNF-α/IL-4, (H) IL-17A/IL-4, (I) IL-2/IL-5, (J) IFN-γ/IL-5, (K) TNF-α/IL-5, (L) IL-17A/IL-5, (M) IL-2/IL-13, (N) IFN-γ/IL-13, (O) TNF-α/IL-13, and (P) IL-17A/IL-13 secretion levels by PBMCs isolated from healthy controls (*n* = 72) and women with RPL (*n* = 99) are displayed. For these non-normally distributed datasets, the Mann–Whitney test was used and median is illustrated. ns, non-significant; *P ≤ 0.05; **P ≤ 0.01; ****P ≤ 0.0001.

Secretion ratios were examined as a marker of pro-/anti-inflammatory balance. TNF-α/IL-5 (*P* = 0.0120), IL-2/IL-13 (*P* = 0.0105), IFN-γ/IL-13 (*P* = 0.0040), and TNF-α/IL-13 (*P* < 0.0001) were augmented in RPL (Fig. 7). These findings suggest a pro-inflammatory signature in non-pregnant women with RPL.

### Robustness of candidate biomarkers and confounder adjustment

Finally, to evaluate robustness of candidate biomarkers, we accounted for major confounding parameters. When comparing cytokine production in the subpopulation of women with idiopathic RPL to that of controls, significant differences identified in the total RPL populations persisted, with the exception of TNF-α^+^/IL-17A^+^ T_H_ cells, TNF-α/IL-10 expression intensity on T_H_ cells, and NK cells’ IL-10 expression intensity (Table 2). The reduction in IL-5 and IL-13 secretion remained significant, exhibiting a higher *P* value. The significant change in IL-8 was, however, lost when examining women with idiopathic RPL, while an additional significant change in IFN-γ and IL-4 was detected (Table 2). Comparing the RPL subpopulations of women with non-idiopathic and idiopathic RPL, the only parameter appearing significantly different was IFN-γ expression intensity on NK cells (Supplementary Table 3).

**Table 2:** Cytokine production comparison between control women and women with idiopathic recurrent pregnancy loss (RPL). Ratios of cytokine production by stimulated T_H_ cells, cytokine production by stimulated NK cells, and protein secretion (pg/mL) in stimulated peripheral blood mononuclear cell cultures of healthy control women and women with RPL and no identifiable risk factors are displayed. Regarding cytokine production, both cytokine-expressing cell percentages and mean expression intensity, assessed via the median fluorescence intensity (MFI), are included. For these non-normally distributed datasets, the Mann–Whitney test was used and median (95% confidence intervals) is depicted. Significant *P *values in the table are highlighted in bold.

Parameter	Controls	Idiopathic RPL	*P* value
IL-2^+^/IL-10^+^ % T_H_	3.21 (1.48, 5.00)	20.06 (6.16, 53.00)	**<0.0001**
IL-2/IL-10 MFI on T_H_	6.64 (4.15, 7.61)	1.25 (0.96, 1.55)	**<0.0001**
IFN-γ^+^/IL-10^+^ % T_H_	1.48 (0.95, 2.88)	19.22 (6.88, 51.45)	**<0.0001**
IFN-γ/IL-10 MFI on T_H_	1.54 (1.05, 2.47)	0.65 (0.52, 1.14)	**<0.0001**
TNF-α^+^/IL-10^+^ % T_H_	7.43 (4.07, 11.30)	69.16 (28.56, 130.40)	**<0.0001**
TNF-α/IL-10 MFI on T_H_	0.48 (0.42, 0.58)	0.27 (0.18, 0.46)	**0.0223**
IL-17A^+^/IL-10^+^ % T_H_	0.08 (0.05, 0.15)	1.28 (0.80, 1.78)	**<0.0001**
IL-17A/IL-10 MFI on T_H_	0.41 (0.36, 0.47)	0.13 (0.10, 0.24)	**<0.0001**
IL-2^+^/IL-17A^+^ % T_H_	25.00 (14.47, 37.13)	20.27 (7.82, 49.17)	0.3434
IL-2/IL-17A MFI on T_H_	16.46 (9.91, 19.39)	10.13 (7.79, 11.70)	**0.0309**
IFN-γ^+^/IL-17A^+^ % T_H_	13.35 (9.26, 16.63)	13.15 (10.04, 21.09)	0.5300
IFN-γ/IL-17A MFI on T_H_	3.83 (3.25, 5.23)	4.39 (3.56, 5.07)	0.5524
TNF-α^+^/IL-17A^+^ % T_H_	72.06 (52.24, 101.80)	45.79 (33.33, 91.82)	0.0960
TNF-α/IL-17A MFI on T_H_	1.27 (0.92, 1.65)	1.65 (1.28, 2.69)	**0.0107**
IFN-γ^+^ % NK	13.30 (8.76, 19.10)	8.17 (4.22, 18.90)	0.2190
IFN-γ MFI on NK	5,976 (4,509, 7,205)	3,117 (2,650, 3,761)	**<0.0001**
TNF-α^+^ % NK	5.56 (3.96, 6.80)	0.97 (0.55, 2.33)	**<0.0001**
TNF-α MFI on NK	1,445 (1,232, 1,735)	2,918 (1,846, 3,855)	**<0.0001**
IL-10^+^ % NK	0.35 (0.24, 0.69)	0.02 (0.00, 0.14)	**<0.0001**
IL-10 MFI on NK	453.00 (406, 504)	379.50 (0, 552)	0.1272
IL-2 secretion	15,039 (6,681, 37,500)	4,369 (1,509, 33,349)	0.1256
IFN-γ secretion	5,850 (3,775, 7,441)	2,450 (1,579, 4,626)	**0.0471**
TNF-α secretion	4,363 (3,657, 5,871)	3,483 (2,040, 4,936)	0.2171
IL-17A secretion	104.50 (63.02, 209.30)	41.06 (17.34, 114.80)	0.0575
IL-10 secretion	183.30 (95.91, 288.30)	90.27 (29.92, 204.10)	0.0687
IL-4 secretion	121.10 (69.98, 246.20)	62.33 (21.96, 120.00)	**0.0305**
IL-5 secretion	130.80 (93.87, 200.80)	46.20 (26.86, 66.23)	**0.0004**
IL-13 secretion	244.70 (142.10, 407.00)	59.74 (31.84, 95.11)	**0.0003**
IL-8 secretion	6,287 (3,889, 9,725)	10,219 (5,064, 14,800)	0.0699
G-CSF secretion	25.16 (16.80, 40.29)	16.80 (16.80, 21.81)	0.1079
GM-CSF secretion	1,384 (745.90, 1,794.00)	736.80 (542.70, 1,187)	0.0797

Following age- and pregnancy history-based normalization, in women with RPL, the decrease in TNF-α^+^ and IL-10^+^ stimulated T_H_ cell percentage and IL-10^+^ unstimulated T_H_ cells was again seen (Supplementary Fig. 8C, 8D, 8 G). Changes in IL-2^+^/IL-10^+^, IFN-γ^+^/IL-10^+^, TNF-α^+^/IL-10^+^, IL-17A^+^/IL-10^+^, and TNF-α^+^/IL-17A^+^ stimulated T_H_ ratios were also seen (Supplementary Fig. 8H, 8I, 8J, 8K, 8L). Reduction in TNF-α^+^ stimulated and unstimulated NK cells persisted after normalization (Supplementary Fig. 8N and 8P). Similarly, an increase in IL-17A, IL-8, and GM-CSF in unstimulated and IL-8 and TNFα/IL-13 concentration in stimulated PBMC cultures also persisted (Supplementary Fig. 8T, 8W, 8Y, 8Z, 8AD). The normalized T cell percentage did not differ between cohorts (Supplementary Fig. 8A). IL-2^+^ and IL-17A^+^ stimulated and TNF-α^+^ unstimulated T_H_ cell percentages, as well as IFN-γ^+^ stimulated and IL-10^+^ stimulated and unstimulated NK cell percentages, were unaltered (Supplementary Fig. 8B, 8E, 8F, 8M, 8O, 8Q). Regarding cytokine concentration in PBMC cultures, IL-5, IL-13, TNF-α/IL-5, IL-2/IL-13, and IFN-γ/IL-13 secretion upon stimulation and IL-2, TNF-α, and IL-5 secretion in the absence of stimulation were also unaltered in RPL (Supplementary Fig. 8R, 8S, 8U, 8V, 8X, 8AA, 8AB, 8AC). Following normalization, IFN-γ^+^/IL-10^+^, TNF-α^+^/IL-10^+^, and IL-17A^+^/IL-10^+^ stimulated T_H_ cell ratios and IL-8 secretion by unstimulated PBMCs produced improved separation between RPL and controls (Supplementary Fig. 8I, J, 8K, 8Y).

The control group included women with and without past successful pregnancies. Subpopulation analysis examining the subgroups of women with and without past live births revealed no differences in production and secretion patterns examined, with the exception of IL-2^+^ T_H_ cell levels following stimulation. When comparing parameters that differ between the total RPL and control cohorts, to the parous-only controls, the significance of IL-17A^+^ T_H_ cell prevalence, IL-8 and IL-13 secretion upon stimulation, and TNF-α secretion in unstimulated cultures was lost. All other parameters remained significantly different (Supplementary Tables 4 and 5).

To account for the influence of age and ethnicity, key parameters were compared between the relevant subpopulations. In control women T_H_ cells’ TNF-α/IL-10 expression intensity and TNF-α^+^ NK cell percentage appeared different between the subgroup of women younger than 30 years of age and the 31–35 subgroup, while IL-2^+^/IL-17A^+^ and IFN-γ^+^/IL-17A^+^ T_H_ cells were altered between the ≤30 and the >40 year old subgroup. However, no age-associated differences were observed in the RPL cohort (Supplementary Table 6). Similarly, in control women of mixed ethnicity, expression intensity of IFN-γ/IL-10 on T_H_ cells was altered compared to Asian and white women and TNF-α/IL-10 T_H_ cell expression intensity was different compared to the other ethnic subpopulations. In addition, IFN-γ/IL-17A T_H_ cell expression intensity was altered between black and white control women. No differences were detected between Asian and white women with RPL (Supplementary Table 7).

## Discussion

T cells are a major factor shaping a pregnancy-supporting environment. Reduced T cells were observed in the periphery of women with RPL, while T_H_, T_C_, and Treg levels were unaltered, despite contradicting findings obtained (Yoo *et al.* 2012, Ghafourian *et al.* 2014, Sereshki *et al.* 2014).

Cytokine levels can provide valuable information regarding the strength and direction of immune responses. It has long been suggested that an anti-inflammatory shift is associated with healthy pregnancy, with a pro-inflammatory shift linked to pregnancy loss (Calleja-Agius & Brincat 2008). Although this shift is thought to primarily affect decidual populations, it is also detectable in circulation pre-conception (Marzi *et al.* 1996, Raghupathy *et al.* 2000).

Historic manuscripts exploring cytokine production in RPL have focused on stimulated T_H_ cells. We additionally incorporated evaluation of NK cell cytokine production as these cells constitute the major pregnancy-associated immune subset, comprising up to 90% of the early-pregnancy uterine lymphocyte population, with their prevalence in circulation being increased in women with RPL (Bagkou Dimakou *et al.* 2025).

Cytokine-producing cell percentages, including IL-2, TNF-α, IL-10, and IL-17A, appeared decreased in RPL, indicating an overall deficiency in cytokine production upon stimulation. Discrepancies between percentage of cytokine-expressing cells and median per cell expression intensity were observed. Although the latter is generally not considered when evaluating cytokine production in RPL, in the present study, this parameter was included, as it contributes to the overall cytokine levels. This approach represents a novel aspect of our analysis.

For instance, TNF-α^+^ and IL-10^+^ T_H_ cell percentages were reduced, but the respective expression intensities were augmented in RPL, indicating a reduced number of cells responding via cytokine production but higher cytokine-producing capacity of individual cells upon activation. Pre-conception IFN-γ production appeared unaltered, in agreement with past findings (Lee *et al.* 2011). Reduced T_H_ cells’ IL-2 production is of particular interest, due to IL-2 being critical for NK maturation, survival, and cytotoxicity (Kim *et al.* 2024). Overall, increased pro-inflammatory cytokine production in circulation was not evident.

IFN-γ/IL-10 and TNF-α/IL-10 production ratios are examined in some RPL clinics to diagnose ‘immunological’ RPL. Interestingly, pro-/anti-inflammatory cytokine-producing T_H_ cell percentages were augmented in RPL, indicating a shift towards T_H_1 and T_H_17 populations, in agreement to the type-1/type-2 hypothesis and clinical biomarkers currently utilized in private practices. However, respective ratios representing pro-/anti-inflammatory cytokine expression intensity were decreased, contradicting the pathogenic relevance of the type-1/type-2 hypothesis and not allowing the formation of conclusion regarding the pro-/anti-inflammatory direction of the response.

Similarly, although TNF-α^+^/IL-17A^+^ cell percentage and IL-2/IL-17A production were reduced in RPL, indicating a shift towards T_H_17 activity, the TNF-α/IL-17A production levels were elevated. It has been suggested that augmented T_H_17 cell/Treg ratio in RPL is linked to vitamin D deficiency (Ji *et al.* 2019), although the mechanism of such association remains undetermined. The IL-17/IL-10 ratio could, thus, be considered as a biomarker in vitamin D trials.

As NK cells are primary pregnancy mediators, their cytokine production was also examined. Upon stimulation, reduced IFN-γ expression intensity was seen in RPL, potentially affecting IFN-γ-mediated HLA induction on embryonic tissues, an NK cell self-regulating mechanism (Carp 2004). Furthermore, TNF-α^+^ NK cells appeared reduced but respective expression levels were elevated, suggesting that in RPL a smaller NK cell number responds to stimulation through the production of TNF-α, synthesizing, however, a larger amount of this cytokine.

NK cells’ IL-10 production appeared reduced in RPL. Stimulation was found to enhance IL-10 expression levels, in the control but not RPL group, indicating defective IL-10 production by circulating NK cells in response to stimulation. In agreement with this hypothesis, decreased IL-4^+^IL-10^+^CD56^bright^ NK cells have been reported in RPL (Fukui *et al.* 2008). In preeclampsia, IL-10 reduction has been speculated to affect Treg proliferation and, thus, establishment of tolerance (Pedroza-Pacheco *et al.* 2013). Interestingly, in contrast to the type-1/type-2 hypothesis, IFN-γ/IL-10 expression levels were reduced in RPL, indicating an overall anti-inflammatory NK contribution. This could affect senescent cell clearance, during decidual remodelling, leading to dead cell accumulation and aberrant inflammation.

Flow cytometry is the go-to option for cytokine testing in RPL clinics. However, considering the discrepancies between the numbers of cytokine-producing cells and per cell cytokine production, no robust conclusion can be formed regarding the overall change in the direction of immune responses in RPL. Cytokine secretion patterns were, thus, investigated as these are expected to be more representative of the *in vivo* cytokine environment.

Augmented TNF-α, IL-17A, IL-8, and GM-CSF and decreased IL-2 and IL-5 release by unstimulated PBMCs were measured in RPL. A reduction in IL-5 plasma levels has also been previously detected (Raghupathy *et al.* 2000). Following stimulation, a reduction in IL-5 and IL-13 secretion and an elevation in IL-8 secretion were also observed in RPL. These changes were reflected in the IL-2/IL-13, IFN-γ/IL-13, TNF-α/IL-13, and TNF-α/IL-5 ratios, which were enhanced in RPL, supporting the hypothesis of a pro-inflammatory shift. The lack of alterations in IFN-γ/IL-10 and TNF-α/IL-10 cytokine secretion ratios indicate that changes in the ratios of cytokine-producing cells, measured by flow cytometry and used in the clinical setting, are possibly counterbalanced by alterations in the per cell cytokine production ratios, not resulting in an altered environment. Hence, secretion assessment or direct evaluation of serum cytokine levels could serve as a more valuable tool for investigation of immune-associated losses.

Interestingly, upon normalization based on the Tommy’s live birth calculator tool, considering influence of age and past pregnancy history, IFN-γ^+^/IL-10^+^, TNF-α^+^/IL-10^+^, IL-17A^+^/IL-10^+^ stimulated T_H_ cell ratios and IL-8 secretion by unstimulated PBMCs displayed a clear separation between cohorts. It should be noted that this only applies to participants between 20 and 45 years of age with a minimum of one past pregnancy, due to the tool’s restrictions. At least in this participant subgroup, when accounting for the major parameters influencing miscarriage risk, our findings highlight a potential diagnostic capacity of cytokine biomarkers.

Our study evaluated the whole cohort of women with RPL, as immune dysregulation could play a contributory role even upon the identification of additional risk factors. Furthermore, pathogenic relevance of RPL risk factors has not been definitively established, except for fetal genetic abnormalities and antiphospholipid syndrome. However, parameters appearing significantly different between RPL and controls, were further assessed in the idiopathic subpopulation, with only TNF-α^+^/IL-17A^+^ T_H_ cell percentage, TNF-α/IL-10 expression intensity on T_H_ cells, IL-10 expression intensity on NK cells, and IL-8 secretion, appearing non-significantly different to controls. This could be explained by the reduced participant numbers in the subpopulation analysis. We took this further to compare women with idiopathic and non-idiopathic RPL, revealing an effect on only NK cells’ IFN-γ expression intensity. These findings highlight a potential but limited contribution of RPL risk factors to certain cytokine biomarkers of interest.

To assess potential impact of inclusion of controls with and without past live births, cytokine parameters appearing significantly different in women with RPL were compared between these subgroups. Only IL-2^+^ T_H_ percentage was found to be reduced in controls with no past birth. Comparing the RPL cohort to parous-only controls, IL-17A^+^ T_H_ cell prevalence, TNF-α secretion, and IL-8 and IL-13 secretion upon stimulation appeared unaltered, indicating that these markers do not constitute strong candidate biomarkers. All other parameters remained significant, indicating no drastic effect of the inclusion of controls with no past live births. In addition, certain parameters appeared influenced by age and ethnicity in the control but not RPL cohort. Low recruitment rates of women with RPL of black and mixed ethnicity did not allow subpopulation analysis of these groups. Thus, further assessment in these participant subpopulations is required.

If not sufficiently regulated *in utero*, the cytokine shifts observed could lead to progesterone resistance, impacting endometrial receptivity, and exacerbated anti-fetal responses (Ewington *et al.* 2019, Blanco-Breindel *et al.* 2023). Although peripheral cytokines are likely not mirroring the local cytokine environment, they may reflect a systemic immune tone affecting both sites. For instance, the type-2 hypothesis has also been described *in utero*, with decidual lymphocytes displaying an even more intense T_H_2 bias than their peripheral counterparts (Wilczyński *et al.* 2003). In addition, TNF-α and IFN-γ polymorphisms have been linked to increased RPL risk (Li *et al.* 2016, Shi *et al.* 2017). Hence, if cytokine alterations observed in women with RPL constitute inherent characteristics, due to genetic variation, peripheral sampling could be used as an indication of systemic imbalance. However, current consensus does not support the extrapolation of conclusion on the *in utero* condition from peripheral data and further studies are required to map peripheral–uterine interactions. Genetic studies may clarify whether the changes measured are an inherent attribute of this condition. Investigation of cytokine levels in paired uterine-peripheral blood samples and cytokine production by uterine and circulating cells will reveal potential markers representative of both sites. Murine and *in vitro* studies interfering with the activity of specific cytokines of interest could add an important mechanistic insight.

In summary, disturbed cytokine expression was observed in non-pregnant women with RPL. Although cytokine fluctuation is expected throughout pregnancy, such findings may indicate a baseline systemic immune dysregulation, not allowing the establishment of an environment capable of supporting healthy pregnancy. Defective pro- and anti-inflammatory cytokine production by NK cells could result in inadequate support to pregnancy establishment. NK cells’ IFN-γ, TNF-α, IL-8, and IL-10 secretion is thought to contribute to spiral artery remodelling and angiogenesis and control trophoblast invasion (Fukui *et al.* 2008, De Oliveira *et al.* 2010, Ticconi *et al.* 2019). Dysregulation of these processes could result in placentation defects, inadequate oxygen and nutrient supply, and thus, fetal demise. The T_H_1 shift in secreted cytokine levels observed in women with RPL could promote the activation of cellular immunity hindering the establishment of tolerance to the fetal semi-allograft. Enhanced secretion of TNF-α and IL-8 could also facilitate thrombosis, threatening pregnancy (Carp 2004). *In utero* cytokine assessment through the different pregnancy stages and miscarriage is required to elucidate the involvement of these molecules in the RPL pathogenic mechanism.

Despite lack of evident pathogenic relevance, peripheral blood cytokine testing for RPL is increasingly popular amongst patients and clinicians (Bagkou Dimakou *et al.* 2022*b*). A blood test predicting RPL risk is highly desirable, as it is less invasive than *in utero* testing and can be performed during pregnancy, when tissue sampling is inaccessible. Observed pre-pregnancy abnormalities could, thus, be assessed as a potential test monitoring immune activity throughout pregnancy progression. The present work largely highlights lack of discrimination between groups. However, upon age- and pregnancy history-based normalization, clear separation was evident in certain participant subgroups indicating significant biomarker potential. Upon validation, novel biomarkers emerging from this study could be used, either alone or in combination with additional risk factors, highlighting women more likely to benefit from immunomodulatory treatment and reassuring women with a low risk of loss.

### Limitations and future research

Recruitment was performed throughout the menstrual cycle, due to the number of participants, allowing for distribution resembling the general population. No information on menstrual timing was collected, which constitutes the major limitation of the current study. Further evaluation of key biomarkers controlling for different menstrual stages could allow standardization of sample collection timing. However, such a test should be robust enough to produce consistent results throughout the cycle as clinical adoption into routine practice may not allow for optimal timing. Time of sample collection was also not standardized and although significant variation was identified between cohorts, an effect of circadian variation is not expected as the majority of samples were collected between ∼12:00 and ∼15:00 h. This variation should, however, be considered upon outcome interpretation.

Difference in alcohol intake is expected as most women with RPL participating in the present study were actively trying to conceive. This could reflect real-world variation, with women with experience of pregnancy loss being more likely to optimize their periconception lifestyle habits. Studies evaluating the influence of alcohol consumption on cytokine-associated measurements are mostly limited on alcohol overconsumption and withdrawal. A study examining the effects of low and moderate consumption reported borderline effects on TNF-α levels (Marques-Vidal *et al.* 2012). Additional demographic parameters potentially affecting cytokine levels that were not accounted for include diet, exercise habits, and psychological stress.

The starting point for the present study was the evaluation of cytokine tests offered in pregnancy loss clinics, primarily in private practices, including IL-2^+^/IL-10^+^, IFN-γ^+^/IL-10^+^, and TNF-α^+^/IL-10^+^ T_H_ cell ratios. We expanded this work to assess IL-17 cytokine expression, as an indication of T_H_17 activity. Due to the inconsistent findings regarding ratios of cytokine-expressing cell percentages vs expression intensity, we employed ELISA. This allowed expansion of markers assessed and evaluation of secreted levels, a more relevant marker regarding impact on immune activity. Hence, certain cytokines assessed via the extended multiplex ELISA panel, such as IL-4, IL-5, and IL-8, where not examined via flow cytometry. It is possible that flow cytometric assessment of these cytokines could reveal significant biomarker potential and, thus, could be addressed in future studies.

PBMC cytokine secretion was assessed by ELISA, due to limited cell availability. To elucidate immune cell contribution to RPL pathogenesis, individual assessment of T and NK cells in future studies would be beneficial. Serum or plasma ELISAs could also be utilized for direct assessment of circulating cytokine levels. Use of additional stimulants, such as K562 cells, embryonic cells, trophoblast extract, and cytokines, could be evaluated to determine whether the variation observed is affected by the stimulation method.

Future steps include multiparametric cytokine assessment and evaluation in relation to participants’ following pregnancy outcomes, with an overarching goal of developing a peripheral blood test that could be used to predict subsequent pregnancy success.

## Supplementary materials



## Declaration of interest

The authors declare no conflict of interest that could be perceived as prejudicing the impartiality of the research reported.

## Funding

We are most thankful for the financial support provided by the Tommy’s Centre for Miscarriage Research (Grant number: 19397).

## Author contribution statement

DBD contributed to data curation, formal analysis, investigation, methodology, visualization, original draft writing, and manuscript review and editing. JT supervised the project and reviewed and edited the manuscript. DL was involved in conceptualization, funding acquisition, methodology, and supervision. AR contributed to conceptualization, funding acquisition, methodology, supervision, and manuscript review and editing.
