# Supplementary material for: Peripheral cytokines as candidate biomarkers for recurrent pregnancy loss
Source: Reprod Fertil. 2026 Jun 19;7(2):RAF250152. doi: 10.1530/RAF-25-0152 (PMC13292982; doi:10.1530/RAF-25-0152)
Supplement: Supplementary file 1 [file supplementary_materials.pdf]

Appendix

Supplementary table 1. Flow cytometry panels. *Antibody targets included to assess A) T cell population and subpopulation prevalence and B) T_H cell and C) NK cell cytokine production are depicted alongside the fluorophore, company, clone, and isotype. APC: allophycocyanin, BV: brilliant violet, CD: cluster of differentiation, FITC: fluorescein isothiocyanate, FoxP3: forkhead box P3, IFN: interferon, IL: interleukin, PE: phycoerythrin, PE-Cy: phycoerythrin-cyanine, PerCP-Cy: peridinin chlorophyll protein-cyanine, TNF: tumor necrosis factor*

**Flow cytometry panels**				
**A) T cell panel**				
**Antibody target**	**Fluorophore**	**Company**	**Clone**	**Isotype**
CD3	APC-H7	BD Biosciences	SK7	Mouse IgG1, κ
CD4	PerCP	BD Biosciences	SK3	Mouse IgG1, κ
CD8	BV510	BD Biosciences	SK1	Mouse IgG1, κ
CD25	FITC	BioLegend	M-A251	Mouse IgG1, κ
CD127	BV421	BD Biosciences	HIL-7R-M21	Mouse IgG1, κ
FoxP3	PE-Cy7	ThermoFisher Scientific	PCH101	Rat IgG2a, κ
**B) T_H cell cytokine pane**				
**Antibody target**	**Fluorophore**	**Company**	**Clone**	**Isotype**
CD3	APC-H7	BD Biosciences	SK7	Mouse IgG1, κ
CD8	BV510	BD Biosciences	SK1	Mouse IgG1, κ
IL-2	PE-Cy7	ThermoFisher Scientific	MQ1-17H12	Rat IgG2a, κ
IL-10	PE	BioLegend	JES3-9D7	Rat IgG1, κ
IL-17A	FITC	Miltenyi Biotec	CZ8-23G1	Mouse IgG1, κ
IFN-γ	BV421	BD Biosciences	4S.B3	Mouse IgG1, κ
TNF-α	APC	ThermoFisher Scientific	MAb11	Mouse IgG1, κ
**C) NK cell cytokine panel**				
**Antibody target**	**Fluorophore**	**Company**	**Clone**	**Isotype**
CD3	APC-H7	BD Biosciences	SK7	Mouse IgG1, κ
CD56	APC	BD Biosciences	NCAM16.2	Mouse IgG2b, κ
CD16	FITC	BD Biosciences	NKP15	Mouse IgG1, κ
IL-10	PE-Cy7	ThermoFisher Scientific	JES3-9D7	Rat IgG1, κ
IFN-γ	BV421	BD Biosciences	4S.B3	Mouse IgG1, κ
TNF-α	PerCP-Cy5.5	ThermoFisher Scientific	MAb11	Mouse IgG1, κ

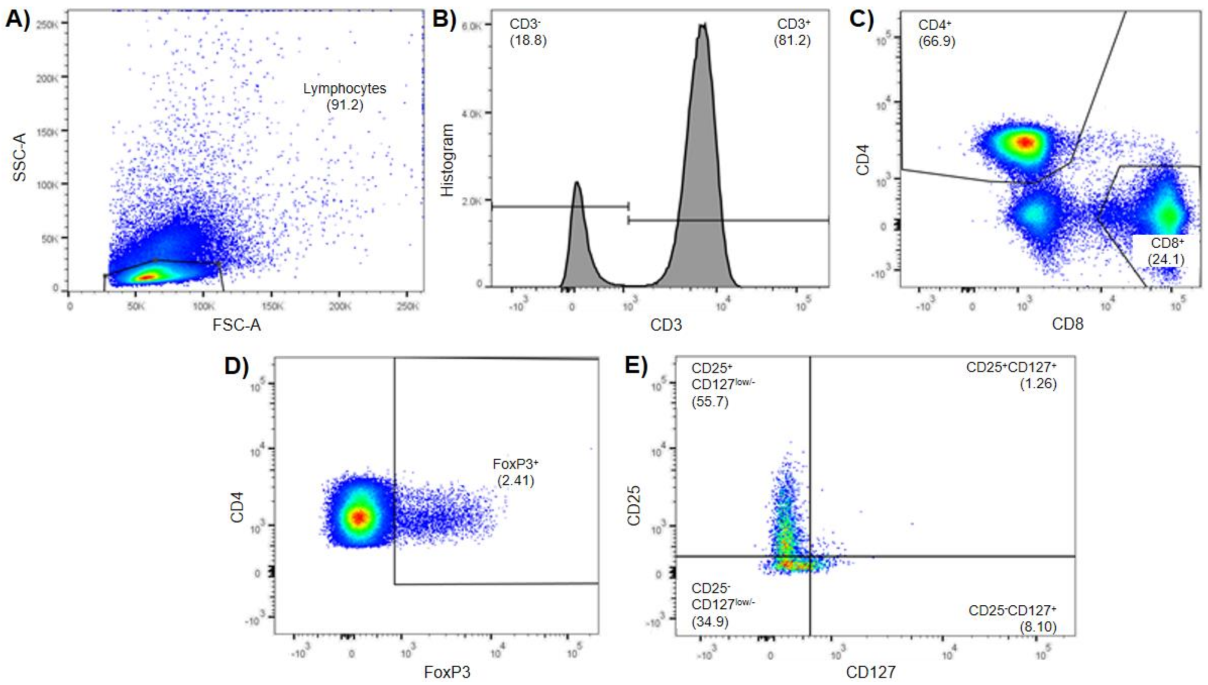

Supplementary figure 1. Flow cytometry gating strategy for the T cell panel. *The singlet population was initially gated on according to its forward scatter area and forward scatter height. Within the singlet gate, A) the lymphocyte population was selected based on its forward (FSC-A) and side (SSC-A) scatter area. In that gate, B) the $CD3^{+}$ T cell population was selected and within that gate, C) the cytotoxic $CD4^{-}CD8^{+}$ and the helper $CD4^{+}CD8^{-}$ subsets were identified. Within the $CD4^{+}CD8^{-}$ population, D) $FoxP3^{+}$ cells were gated on, and E) $CD25^{+}CD127^{low/-}$ regulatory T cells were subsequently selected.*

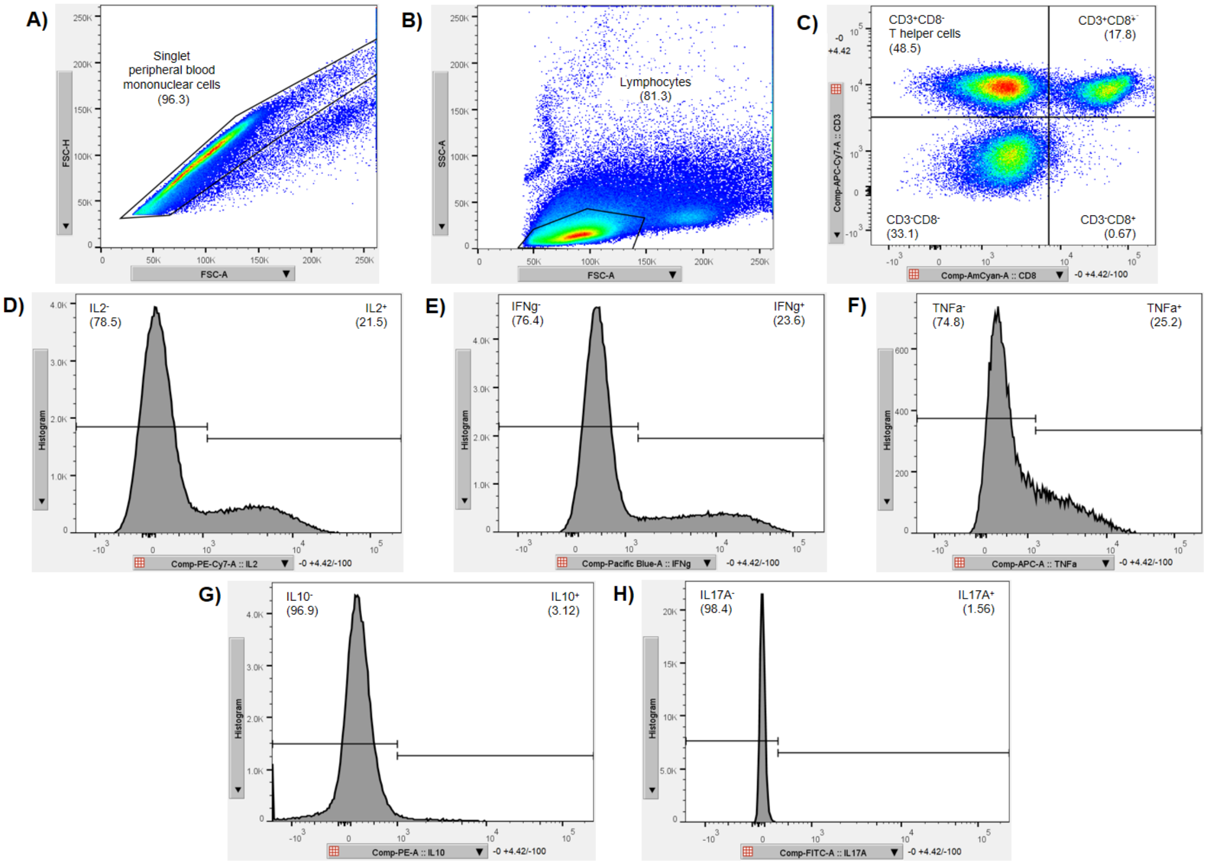

Supplementary figure 2. Flow cytometry gating strategy for T helper cell cytokine panel. *A) The singlet population was gated on according to the forward scatter area (FSC-A) and forward scatter height (FSC-H). B) In the singlet gate lymphocytes were selected based on their forward (FSC-A) and side (SSC-A) scatter area. C) In that gate, $CD3^{+}CD8^{-}$ T helper cells were selected and in that gate D) IL-2, E) IFN-γ, F) TNF-α, G) IL-10, and H) IL-17A cytokine expression was assessed.*

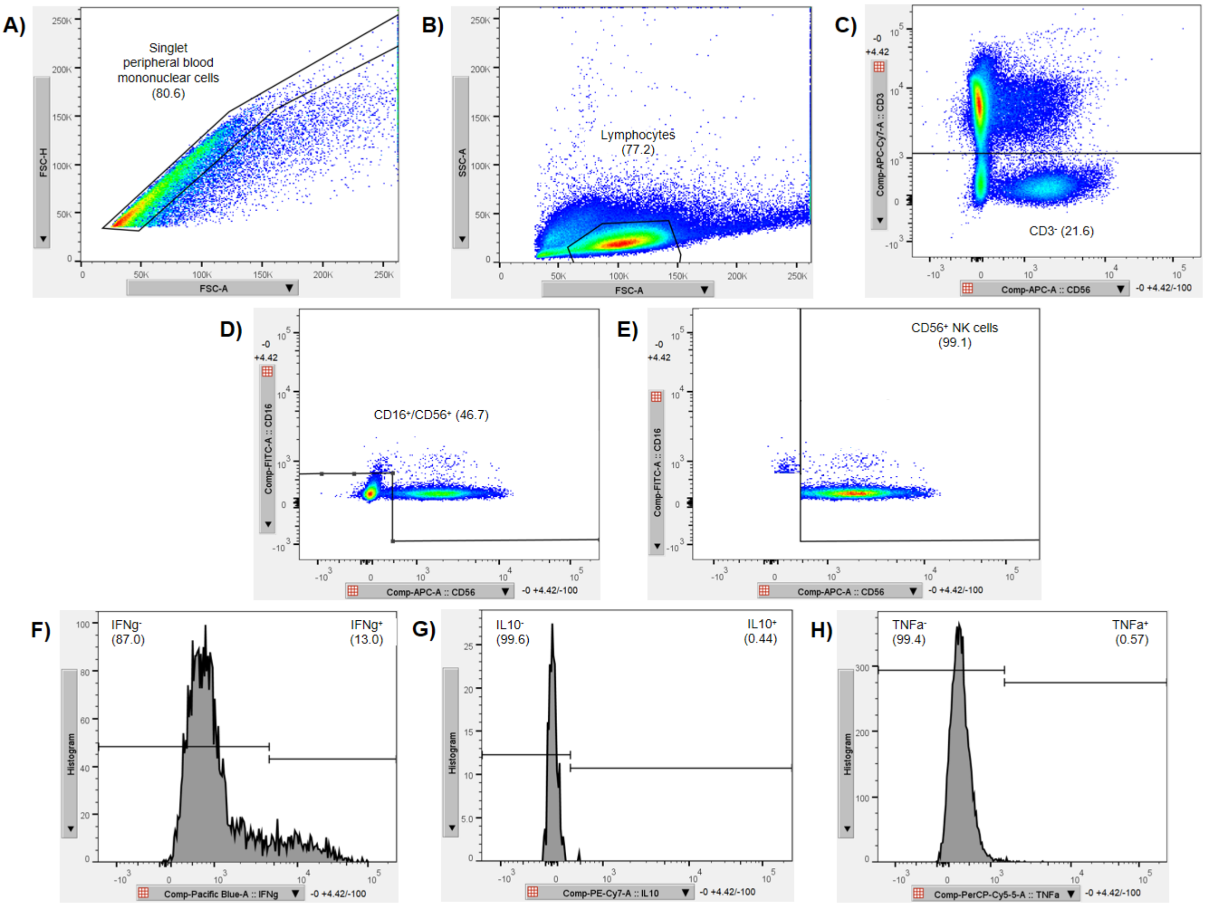

Supplementary figure 3. Flow cytometry gating strategy for Natural Killer cell cytokine panel.

A) The singlet population was gated on according to the forward scatter area (FSC-A) and forward scatter height (FSC-H). B) In the singlet gate, lymphocytes were gated on based on their forward (FSC-A) and side (SSC-A) scatter area. C) In that gate CD3$^-$ cells were selected and D) within that population CD56$^+$ and/or CD16$^+$ cells were gated on. E) In the CD56$^+$/CD16$^+$ population, the total CD56$^+$ NK cell population was selected and within these gates F) IFN-γ, G) IL-10, and H) TNF-α expression was assessed.

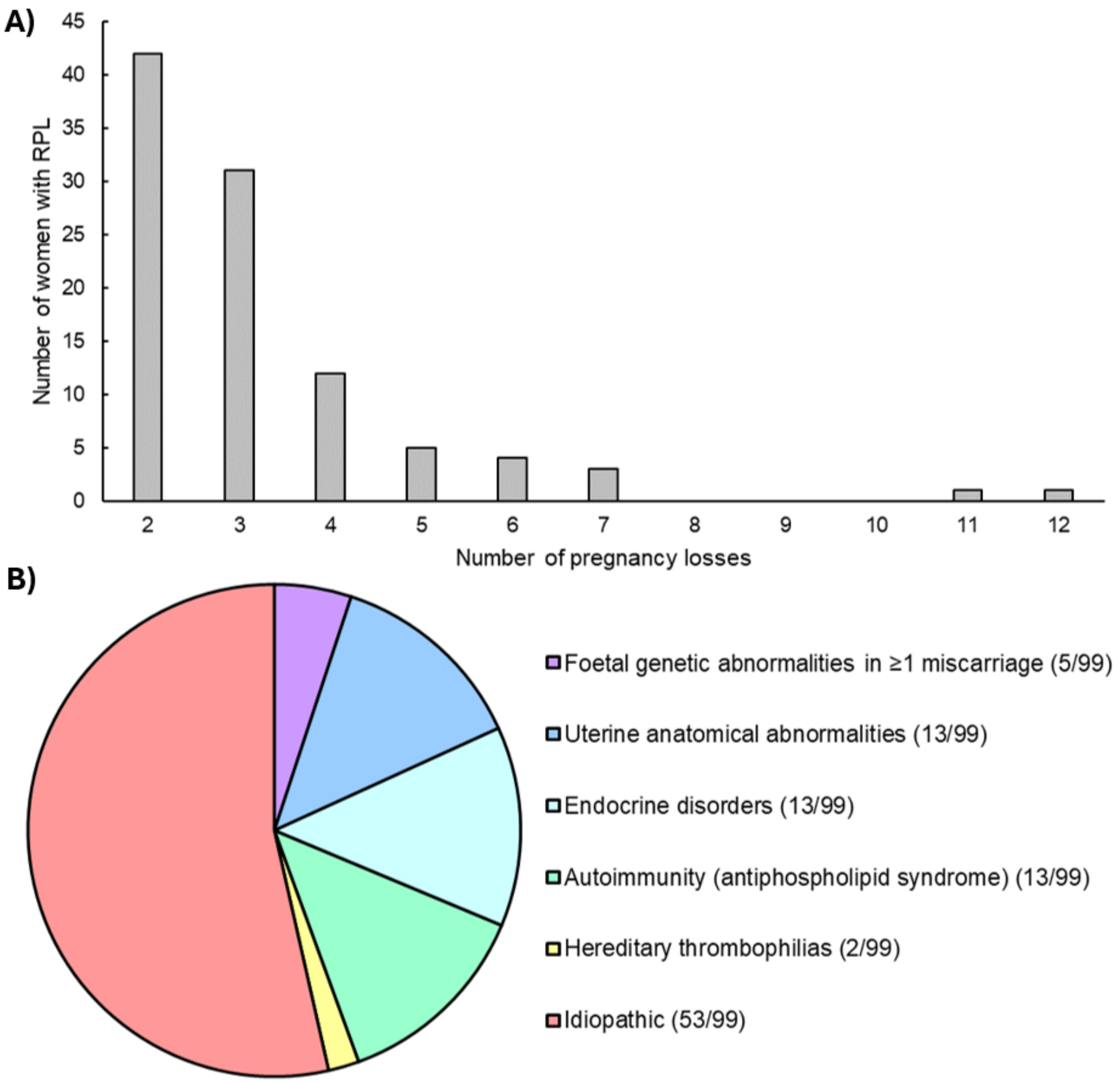

Supplementary figure 4. Number of pregnancy losses and underlying pathologies identified in the recurrent pregnancy loss (RPL) cohort. *Women in the RPL cohort (N=99), subcategorised according to A) the number of their past losses and B) RPL-associated pathologies or their absence (idiopathic RPL), are depicted.*

Supplementary table 2. Cytokine production by unstimulated and stimulated T_H and NK cells and protein concentration (pg/mL) in unstimulated and stimulated peripheral blood mononuclear cell (PBMC) cultures derived from women with recurrent pregnancy loss (RPL) and controls. *IL-2^{+}, IFN-γ^{+}, TNF-α^{+}, IL-10^{+}, and IL-17A^{+} CD4^{+} T_H cell percentages and expression intensity of IL-2, IFN-γ, TNF-α, IL-10, and IL-17A on individual CD4^{+} T_H cells, represented by the median fluorescence intensity (MFI), in healthy controls (N=76) and women with RPL (N=82) in the*

absence and following stimulation with phorbol 12-myristate 13-acetate and ionomycin is depicted. IFN-γ$^+$, TNF-α$^+$, and IL-10$^+$ NK cell percentages and expression intensity of IFN-γ, TNF-α, and IL-10 on individual NK cells, represented by the MFI, in healthy controls (N=71) and women with RPL (N=84) in the absence and following stimulation is displayed. The levels of IL-2, IL-10, IL-8, IFN-γ, IL-4, G-CSF, TNF-α, IL-5, GM-CSF, IL-17A, and IL-13 secretion by PBMCs isolated from healthy controls (N=72) and women with RPL (N=99) in the absence and following stimulation is also displayed. For these non-normally distributed datasets, Wilcoxon matched-pairs signed rank test was used and median alongside 95% confidence interval are illustrated.

Parameter	Controls			RPL		
	Unstimulated	Stimulated	p value	Unstimulated	Stimulated	p value
**IL-2$^+$ % T_H**	0.02 (0.01, 0.03)	19.25 (13.80, 25.60)	**<0.0001**	0.02 (0.1, 0.02)	12.45 (8.49, 17.90)	**<0.0001**
**IL-2 MFI on T_H**	2554 (2076, 3448)	6169 (5082, 8543)	**<0.0001**	2544 (1976, 3095)	5109 (4352, 5862)	**<0.0001**
**IFN-γ$^+$ % T_H**	0.32 (0.20, 0.61)	9.37 (6.75, 12.60)	**<0.0001**	0.36 (0.14, 0.50)	6.73 (5.95, 9.97)	**<0.0001**
**IFN-γ MFI on T_H**	1161 (895, 1400)	2078 (1674, 3064)	**<0.0001**	1409 (1232, 1576)	2637 (1877, 3420)	**<0.0001**
**TNF-α$^+$ % T_H**	13.40 (9.52, 16.40)	52.35 (44.00, 61.30)	**<0.0001**	3.13 (1.90, 5.51)	29.85 (24.40, 37.30)	**<0.0001**
**TNF-α MFI on T_H**	248 (229, 266)	562.50 (434, 720)	**<0.0001**	491.50 (394, 642)	896.50 (694, 1237)	**<0.0001**
**IL-10$^+$ % T_H**	0.82 (0.24, 1.89)	6.70 (3.57, 11.90)	**<0.0001**	0.01 (0.00, 0.04)	0.43 (0.24, 0.80)	**<0.0001**
**IL-10 MFI on T_H**	828 (665, 950)	1073 (868, 1371)	**<0.0001**	832.50 (531, 1297)	3466 (2144, 4801)	**<0.0001**
**IL-17A$^+$ % T_H**	0.18 (0.11, 0.29)	0.67 (0.51, 0.88)	**<0.0001**	0.11, (0.07, 0.20)	0.46 (0.37, 0.59)	**<0.0001**
**IL-17A MFI on T_H**	313 (286, 367)	416 (329, 593)	**<0.0001**	368.50 (322, 394)	433 (362, 493)	**<0.0001**
**IFN-γ$^+$ % NK**	0.91 (0.61, 1.54)	13.30 (8.76, 19.10)	**<0.0001**	1.29 (0.63, 2.58)	8.91 (6.09, 18.90)	**<0.0001**
**IFN-γ MFI on NK**	2529 (2087, 3058)	5976 (4509, 7205)	**<0.0001**	2676 (2091, 2946)	3205 (2928, 3777)	**<0.0001**
**TNF-α$^+$ % NK**	1.50 (0.43, 2.78)	5.56 (3.96, 6.80)	**0.0046**	0.11 (0.03, 0.22)	1.11 (0.65, 2.33)	**<0.0001**
**TNF-α MFI on NK**	1121 (950, 1270)	1445 (1232, 1735)	**0.0054**	1274 (892, 1670)	2832 (2132, 3345)	**<0.0001**
**IL-10$^+$ % NK**	0.05 (0.00, 0.23)	0.35 (0.24, 0.69)	0.0797	0.00 (0.00, 0.02)	0.04 (0.00,0.17)	0.1254
**IL-10 MFI on NK**	336 (0.00, 433)	453 (406, 504)	**0.0012**	0.00 (0.00, 240)	352.50 (0.00, 497)	0.1725
**IL-2 secretion**	9.16 (7.74, 11.55)	15039 (6681, 37500)	**<0.0001**	7.42 (6.37, 8.79)	6986 (2705, 34854)	**<0.0001**
**IL-10 secretion**	74.84 (4.17, 823)	183.30 (95.91, 288.30)	0.1800	263.10 (121, 623.40)	100.60 (61.99, 267.60)	**0.0069**
**IL-8 secretion**	3553 (2128, 5060)	6287 (3889, 9725)	**0.0090**	7405 (5943, 12669)	10219 (6968, 13477)	0.3113
**IFN-γ secretion**	12.18 (12.18, 12.18)	5850 (3775, 7441)	**<0.0001**	12.18 (12.18, 12.18)	3237 (2335, 4626)	**<0.0001**
**IL-4 secretion**	5.99 (4.20, 7.57)	121.10 (69.98, 246.20)	**<0.0001**	12.65 (4.37, 12.65)	83.12 (44.55, 129.20)	**<0.0001**
**G-CSF secretion**	85.91 (16.80, 401)	25.16 (16.80, 40.29)	**<0.0001**	303 (101.60, 618.80)	16.80 (16.80, 21.81)	**<0.0001**
**TNF-α secretion**	523.80 (53.04, 2966)	4363 (3657, 5871)	**0.0002**	3332 (1908, 6506)	4038 (2477, 5329)	0.1916
**IL-5 secretion**	21.80 (17.40, 27.75)	130.80 (93.87, 200.80)	**<0.0001**	15.84 (11.31, 15.84)	53.86 (36.42, 68.84)	**<0.0001**
**GM-CSF secretion**	56.94 (18.43, 104.60)	1384 (745.90, 1794)	**<0.0001**	146.90 (72.67, 299.10)	869.90 (657.50, 1200)	**<0.0001**
**IL-17A secretion**	2.81 (1.93, 2.81)	104.50 (63.02, 209.30)	**<0.0001**	2.81 (2.81, 2.81)	57.02 (30.50, 114.80)	**<0.0001**
**IL-13 secretion**	4.39 (4.39, 4.39)	244.70 (142.10, 407)	**<0.0001**	4.39 (4.39, 4.39)	72.35 (38.19, 128.30)	**<0.0001**

Percentage of cytokine-expressing CD4$^+$ T cells in the absence of stimulation

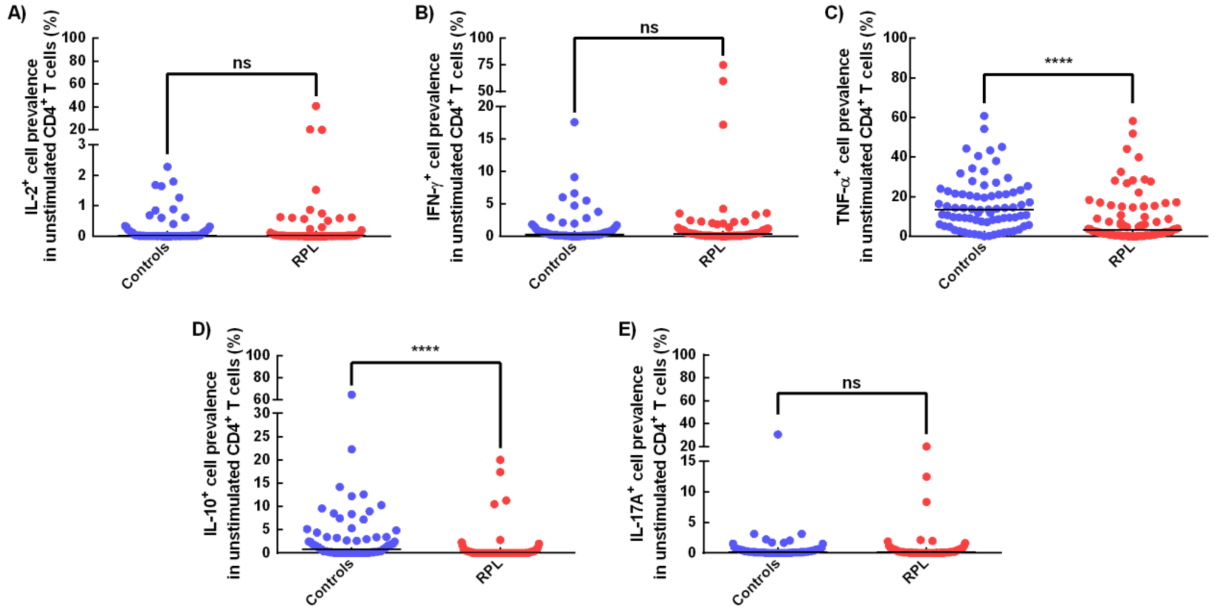

Cytokine expression intensity on individual CD4$^+$ T cells in the absence of stimulation

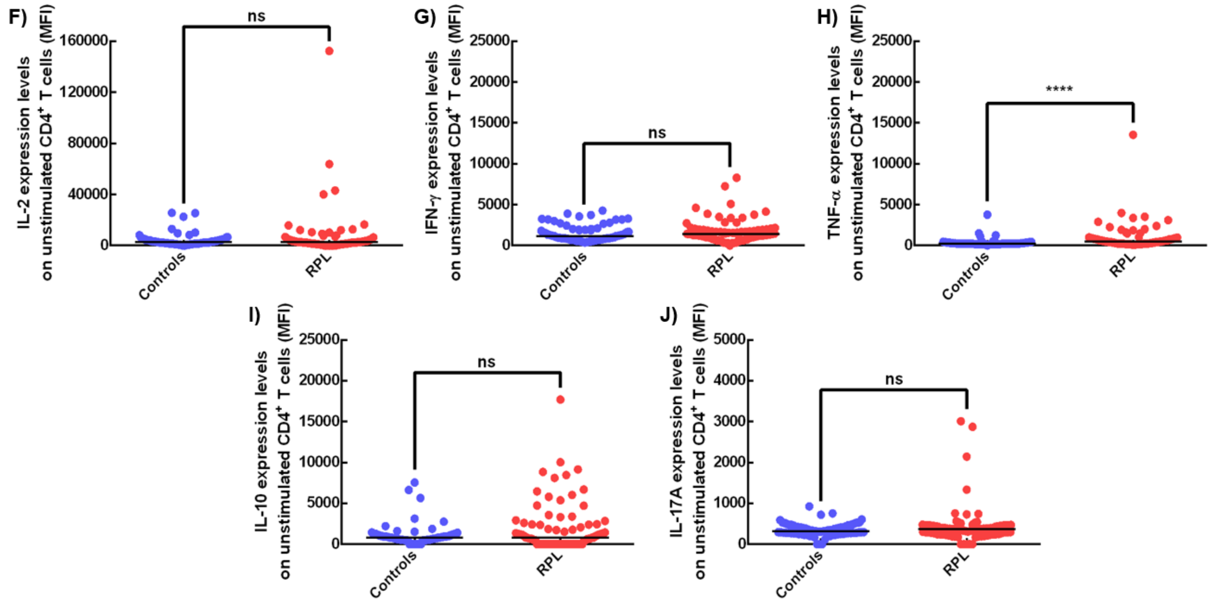

Supplementary figure 5. Cytokine production by unstimulated T_H cells of women with recurrent pregnancy loss (RPL) and controls. *A) IL-2$^+$, B) IFN-γ$^+$, C) TNF-α$^+$, D) IL-10$^+$, and E) IL-17A$^+$ CD4$^+$ T_H cell percentages and expression intensity of F) IL-2, G) IFN-γ, H) TNF-α, I) IL-10, and J) IL-17A on individual CD4$^+$ T_H cells, represented by the median fluorescence intensity (MFI), in healthy controls (N=76) and women with RPL (N=82) are illustrated. For these non-normally distributed datasets, Mann-Whitney test was used and median depicted. ns: non-significant, ****: $p \leq 0.0001$*

Percentage of cytokine-expressing NK cells in the absence of stimulation

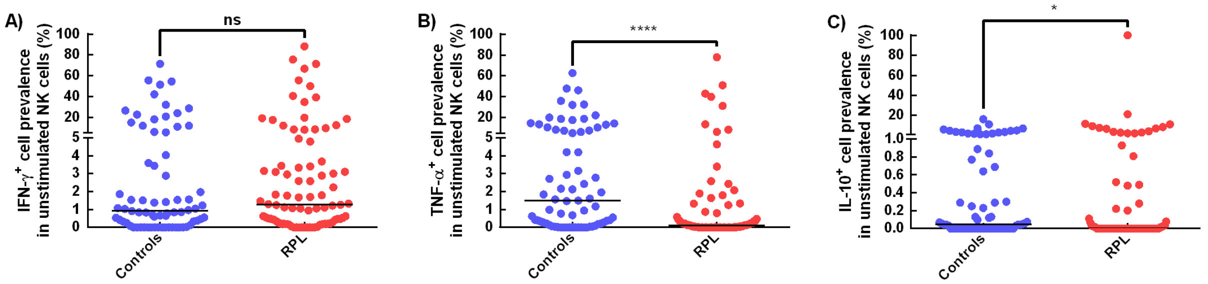

Cytokine expression intensity on individual NK cells in the absence of stimulation

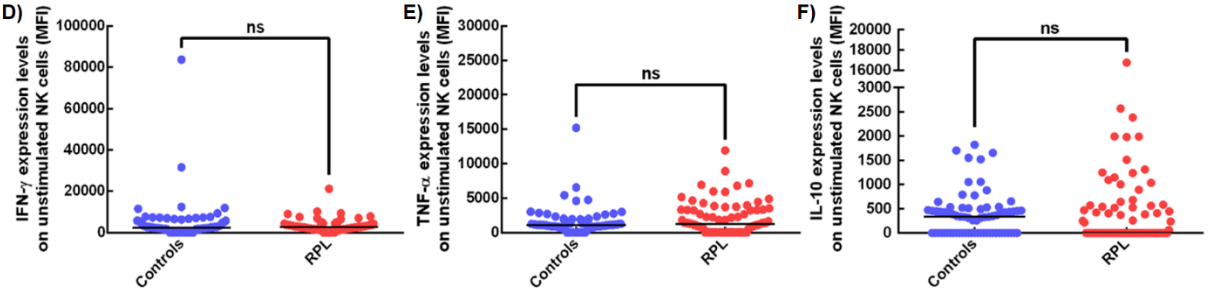

Supplementary figure 6. Cytokine production by unstimulated NK cells of women with recurrent pregnancy loss (RPL) and controls. *A) IFN-γ^{+}, B) TNF-α^{+}, and C) IL-10^{+} NK cell percentages and expression intensity of D) IFN-γ, E) TNF-α, and F) IL-10 on individual NK cells, represented by the median fluorescence intensity (MFI), in healthy controls (N=71) and women with RPL (N=84) are illustrated. For these non-normally distributed datasets, Mann-Whitney test was used and median depicted. ns: non-significant, *: p≤0.05, ****: p≤0.0001*

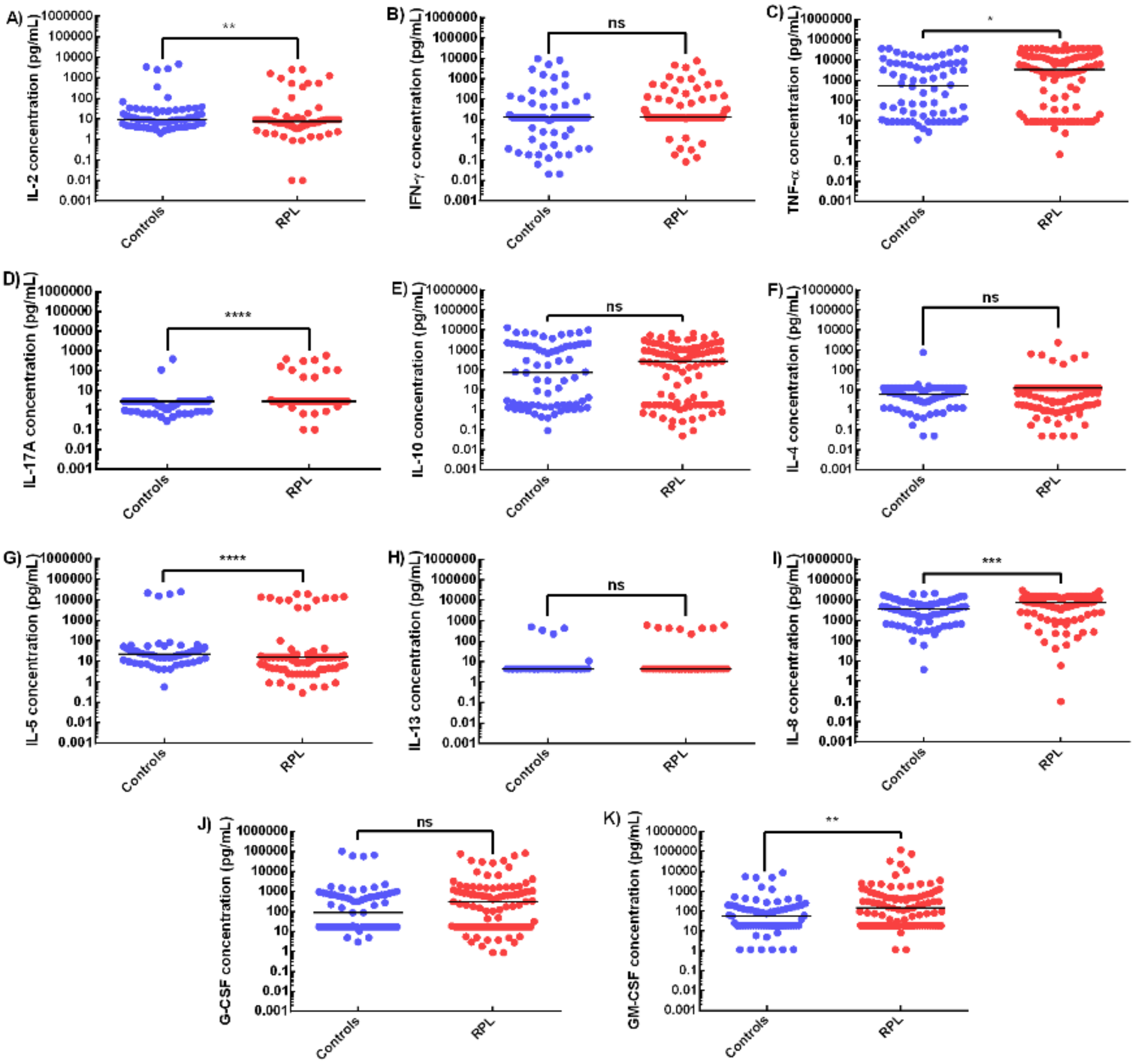

Supplementary figure 7. Protein concentration (pg/mL) in unstimulated peripheral blood mononuclear cell (PBMC) cultures derived from women with recurrent pregnancy loss (RPL) and controls. *The levels of A) IL-2, B) IFN-γ, C) TNF-α, D) IL-17A, E) IL-10, F) IL-4, G) IL-5, H) IL-13, I) IL-8, J) G-CSF, and K) GM-CSF secretion by PBMCs isolated from healthy controls (N=72) and women with RPL (N=99) is depicted. For these non-normally distributed datasets, Mann-Whitney test was performed and median illustrated. ns: non-significant, *: p≤0.05, **: p≤0.01, ***: p≤0.001, ****: p≤0.0001*

Supplementary table 3. Cytokine production comparison between women with non-idiopathic and women with idiopathic recurrent pregnancy loss (RPL). *Ratios of cytokine production by*

stimulated T_H cells, cytokine production by stimulated NK cells, and protein secretion (pg/mL) in stimulated peripheral blood mononuclear cell cultures from women with RPL with and without identifiable risk factors are depicted. Regarding cytokine production, both cytokine-expressing cell percentages and mean expression intensity, assessed via the median fluorescence intensity (MFI), are included. For these non-normally distributed datasets, Mann-Whitney test was performed and median (95% confidence intervals) displayed.

Parameter	Non-idiopathic RPL	Idiopathic RPL	p value
**IL-2$^+$/IL-10$^+$ % T_H**	20.32 (4.66, 46.10)	20.06 (6.16, 53.00)	0.8642
**IL-2/IL-10 MFI on T_H**	1.43 (1.04, 2.82)	1.25 (0.96, 1.55)	0.5141
**IFN-γ$^+$/IL-10$^+$ % T_H**	16.26 (5.75, 26.26)	19.22 (6.88, 51.45)	0.3103
**IFN-γ/IL-10 MFI on T_H**	0.95 (0.53, 1.64)	0.65 (0.52, 1.14)	0.2055
**TNF-α$^+$/IL-10$^+$ % T_H**	50.05 (20.53, 131.80)	69.16 (28.56, 130.40)	0.3866
**TNF-α/IL-10 MFI on T_H**	0.38 (0.19, 0.46)	0.27 (0.18, 0.46)	0.7287
**IL-17A$^+$/IL-10$^+$ % T_H**	0.98 (0.48, 1.59)	1.28 (0.80, 1.78)	0.2326
**IL-17A/IL-10 MFI on T_H**	0.21 (0.10, 0.36)	0.13 (0.10, 0.24)	0.6671
**IL-2$^+$/IL-17A$^+$ % T_H**	28.09 (16.73, 40.48)	20.27 (7.82, 49.17)	0.6255
**IL-2/IL-17A MFI on T_H**	10.34 (8.01, 11.41)	10.13 (7.79, 11.70)	0.8208
**IFN-γ$^+$/IL-17A$^+$ % T_H**	14.16 (8.59, 18.33)	13.15 (10.04, 21.09)	0.6671
**IFN-γ/IL-17A MFI on T_H**	5.39 (3.56, 7.77)	4.39 (3.56, 5.07)	0.3472
**TNF-α$^+$/IL-17A$^+$ % T_H**	54.36 (32.12, 85.26)	45.79 (33.33, 91.82)	0.8769
**TNF-α/IL-17A MFI on T_H**	1.67 (1.46, 2.70)	1.65 (1.28, 2.69)	0.9521
**IFN-γ$^+$ % NK**	17.30 (5.37, 22.70)	8.17 (4.22, 18.90)	0.4867
**IFN-γ MFI on NK**	3526 (3016, 5652)	3117 (2650, 3761)	**0.0449**
**TNF-α$^+$ % NK**	1.76 (0.52, 3.50)	0.97 (0.55, 2.33)	0.6372
**TNF-α MFI on NK**	2369 (1924, 3339)	2918 (1846, 3855)	0.5656
**IL-10$^+$ % NK**	0.11 (0.00, 0.39)	0.02 (0.00, 0.14)	0.1494
**IL-10 MFI on NK**	340 (0, 558)	379.50 (0, 552)	0.9760
**IL-2 secretion**	15495 (2705, 37500)	4369 (1509, 33349)	0.2177
**IFN-γ secretion**	4028 (1986, 6922)	2450 (1579, 4626)	0.3509
**TNF-α secretion**	4199 (2389, 9663)	3483 (2040, 4936)	0.2960
**IL-17A secretion**	57.02 (17.35, 200.30)	41.06 (17.34, 114.80)	0.4807
**IL-10 secretion**	212.50 (50.58, 415.80)	90.27 (29.92, 204.10)	0.2682
**IL-4 secretion**	129.20 (43.75, 297.00)	62.33 (21.96, 120.00)	0.2218
**IL-5 secretion**	63.79 (31.07, 112.40)	46.20 (26.86, 66.23)	0.3273
**IL-13 secretion**	124.50 (34.82, 276.30)	59.74 (31.84, 95.11)	0.1309
**IL-8 secretion**	10001 (4577, 14800)	10219 (5064, 14800)	0.9156
**G-CSF secretion**	20.21 (16.80, 45.48)	16.80 (16.80, 21.81)	0.3910
**GM-CSF secretion**	1188 (579.30, 1460.00)	736.80 (542.70, 1187.00)	0.2575

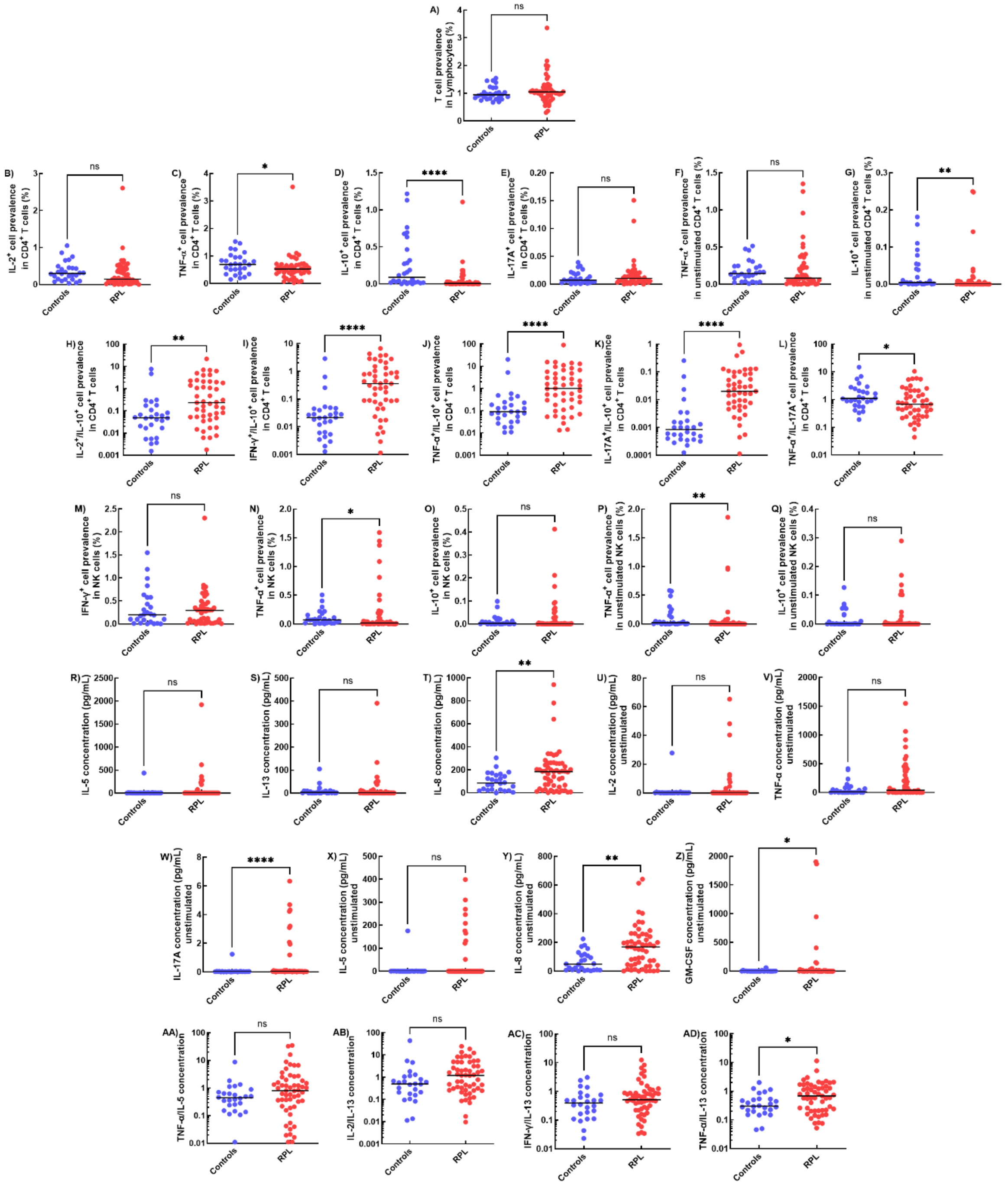

Supplementary figure 8. T cell population prevalence, T and NK cell cytokine production, and cytokine concentration (pg/mL) in peripheral blood mononuclear cell (PBMC) cultures in control women and women with recurrent pregnancy loss (RPL), normalized based on probability of live birth, calculated according to age and pregnancy history. *Probability of birth (%) was calculated according to age and outcomes of the 5 more recent pregnancies, using the Tommy's live birth calculator. Only participants between 20 and 45 years of age, with at least one past pregnancy, and known order of pregnancy outcomes were included. Ratio normalization*

*was performed according to the following equation: (normalized prevalence) = [non-normalized prevalence (%) / probability of birth (%)]. Normalized A) T cell prevalence in the peripheral blood lymphocyte population, B) IL-2$^+$, C) TNF-α$^+$, D) IL-10$^+$, and E) IL-17A$^+$ stimulated and F) TNF-α$^+$ and G) IL-10$^+$ unstimulated T_H cell percentages, H) IL-2$^+$/IL-10$^+$, I) IFN-γ$^+$/IL-10$^+$, J) TNF-α$^+$/IL-10$^+$, K) IL-17A$^+$/IL-10$^+$, and L) TNF-α$^+$/IL-17A$^+$ stimulated T_H cell ratio, M) IFN-γ$^+$, N) TNF-α$^+$, and O) IL-10$^+$ and P) TNF-α$^+$ and Q) IL-10$^+$ unstimulated T_H cell percentages, levels of R) IL-5, S) IL-13, and T) IL-8 secretion by stimulated PBMCs, levels of U) IL-2, V) TNF-α, W) IL-17A, X) IL-5, Y) IL-8, and Z) GM-CSF secretion by unstimulated PBMCs, and ratios of AA) TNF-α/IL-5, AB) IL-2/IL-13, AC) IFN-γ/IL-13, and AD) TNF-α/IL-13 secretion by stimulated PBMCs in controls (N=28) and women with RPL (N=55) is depicted. For these non-normally distributed datasets, Mann-Whitney test was used and median shown. ns: non-significant, *: p≤0.05, **: p≤0.01, ****: p≤0.0001*

Supplementary table 4. Cytokine production in control women with past births (parous), in comparison to control women without past births (nulliparous) and women with recurrent pregnancy loss (RPL). *Cytokines whose production, as examined by flow cytometry, appeared significantly different between control women and women with RPL were assessed in control parous women (N=33), compared to control nulliparous women (N=44) and the RPL population. The percentage of cytokine-expressing cells in the stimulated and unstimulated* T_H *and NK cell populations is included. Normally distributed datasets (TNF-*α^+ *%* T_H*): Unpaired t test performed, mean and standard deviation depicted. Non-normally distributed datasets: Mann-Whitney test used, median and 95% confidence interval included.*

**Parameter**		**Controls**		**RPL**	**p value**	
		**Nulliparous**	**Parous**		**Nulliparous vs parous controls**	**RPL vs parous controls**
**Stimulated**	**IL-2$^+$ % T_H**	15.00 (11.70, 20.80)	25.45 (14.90, 37.20)	12.45 (8.49, 17.90)	**0.0246**	**0.0005**
	**TNF-α$^+$ % T_H**	47.85 (19.15)	55.03 (21.61)	33.44 (21.17)	0.1304	**<0.0001**
	**IL-10$^+$ % T_H**	7.54 (4.13, 15.20)	3.97 (1.99, 17.40)	0.43 (0.24, 0.80)	0.4881	**<0.0001**
	**IL-17A$^+$ % T_H**	0.72 (0.52, 0.98)	0.60 (0.28, 0.89)	0.46 (0.37, 0.59)	0.1941	0.5016
	**TNF-α$^+$ % NK**	5.05 (5.79, 13.87)	5.87 (3.25, 10.20)	1.11 (0.65, 2.33)	0.8919	**0.0010**
	**IL-10$^+$ % NK**	0.35 (0.27, 0.68)	0.45 (0.09, 1.04)	0.04 (0.00, 0.17)	0.9884	**0.0148**
**Unstimulated**	**TNF-α$^+$ % T_H**	14.80 (9.68, 20.60)	10.50 (7.50, 14.60)	3.56 (1.91, 5.76)	0.1257	**0.0021**
	**IL-10$^+$ % T_H**	1.66 (0.33, 2.48)	0.31 (0.07, 1.62)	0.01 (0.00, 0.04)	0.1431	**0.0002**
	**TNF-α$^+$ % NK**	0.77 (0.23, 2.94)	2.05 (0.68, 10.50)	0.11 (0.03, 0.22)	0.2563	**<0.0001**
	**IL-10$^+$ % NK**	0.05 (0.00, 0.25)	0.06 (0.00, 0.89)	0.00 (0.00, 0.02)	0.4126	**0.0447**

Supplementary table 5. Cytokine secretion in control women with past births (parous), in comparison to control women without past births (nulliparous) and women with recurrent pregnancy loss (RPL). *Cytokines whose secretion, as evaluated via ELISA, appeared significantly different between control women and women with RPL were assessed in control parous women (N=30), compared to control nulliparous women (N=42) and the RPL population. For these non-normally distributed datasets, Mann-Whitney test was used, with median and 95% confidence interval depicted.*

**Parameter**		**Controls**		**RPL**	**p value**	
		**Nulliparous**	**Parous**		**Nulliparous vs parous controls**	**RPL vs parous controls**
**Stimulated**	**IL-13**	233.10 (142.10, 428.10)	263.90 (85.23, 494.30)	72.35 (38.19, 128.30)	0.6620	0.0852
	**IL-5**	111.50 (71.01, 220.10)	162.50 (64.60, 257.30)	53.86 (36.42, 68.84)	0.7963	**0.0359**
	**IL-8**	5754 (2653, 10934)	6573 (1839, 9793)	10219 (6968, 13477)	0.9028	0.1002
**Unstimulated**	**GM-CSF**	21.34 (18.43, 126.60)	67.92 (18.43, 116.60)	146.90 (72.67, 299.10)	0.7614	**0.0388**

	**IL-17A**	2.57 (1.55, 2.81)	2.81 (2.32, 2.81)	2.81 (2.81, 2.81)	0.1983	**0.0011**
	**IL-2**	8.77 (6.83, 10.91)	11.37 (6.83, 17.56)	7.42 (6.37, 8.79)	0.5797	**0.0123**
	**IL-5**	22.01 (16.38, 32.79)	20.79 (15.84, 32.79)	15.84 (11.31, 15.84)	0.9434	**0.0008**
	**IL-8**	4652 (2497, 6672)	2212 (658.70, 4702)	7405 (5943, 12669)	0.2875	**0.0016**
	**TNF-α**	287.80 (31.80, 3464)	620.40 (30.09, 4428)	3332 (1908, 6506)	0.8891	0.0641

Supplementary table 6. Cytokine production in control women and women with recurrent pregnancy loss (RPL), belonging in different age groups. *T_H cell ratios and NK cell cytokine production upon stimulation, assessed via the number of cytokine-producing cells and the per cell production represented via the medial fluorescence intensity (MFI), are displayed. For these non-normally distributed datasets, Kruskal-Wallis test was performed, with median and 95% confidence interval included.*

Parameter	≤30 years	31-35 years	36-40 years	>40 years	p value
**Controls**					
**IL-2$^+$/IL-10$^+$ % T_H**	1.53 (0.75, 4.66)	2.55 (0.52, 9.70)	13.10 (0.42, 1400)	4.41 (1.06, 17.93)	0.0643
**IL-2/IL-10 MFI on T_H**	5.24 (3.37, 7.61)	7.42 (3.62, 19.36)	4.08 (0.43, 18.11)	6.99 (3.80, 8.81)	0.2991
**IFN-γ$^+$/IL-10$^+$ % T_H**	1.00 (0.43, 3.19)	1.18 (0.30, 3.50)	3.67 (0.23, 204.00)	1.66 (1.02, 5.43)	0.2450
**IFN-γ/IL-10 MFI on T_H**	1.26 (0.93, 2.47)	2.99 (1.05, 5.09)	1.05 (0.42, 4.66)	1.96 (1.02, 5.82)	0.1508
**TNF-α$^+$/IL-10$^+$ % T_H**	7.22 (3.41, 9.69)	4.82 (2.14, 20.11)	32.22 (1.42, 4833)	11.31 (2.31, 20.71)	0.2100
**TNF-α/IL-10 MFI on T_H**	0.44 (0.33, 0.49)	0.80 (0.46, 2.68)	0.31 (0.08, 0.91)	0.61 (0.39, 1,15)	**0.0032**
**IL-17A$^+$/IL-10$^+$ % T_H**	0.09 (0.03, 0.26)	0.05 (0.03, 0.15)	0.25 (0.03, 18.00)	0.06 (0.04, 0.17)	0.4060
**IL-17A/IL-10 MFI on T_H**	0.40 (0.32, 0.48)	0.41 (0.33, 0.96)	0.55 (0.07, 1.40)	0.40 (0.32, 0.53)	0.7213
**IL-2$^+$/IL-17A$^+$ % T_H**	13.71 (10.75, 20.26)	62.93 (13.01, 88.92)	51.56 (7.48, 140.00)	42.42 (20.55, 231.50)	**0.0022**
**IL-2/IL-17A MFI on T_H**	12.84 (8.46, 19.65)	17.61 (4.66, 29.46)	11.03 (0.79, 44.63)	19.39 (6.65, 39.14)	0.7508
**IFN-γ$^+$/IL-17A$^+$ % T_H**	10.27 (6.69, 15.06)	23.92 (9.11, 42.80)	10.98 (4.20, 75.20)	19.05 (10.00, 46.67)	**0.0156**
**IFN-γ/IL-17A MFI on T_H**	3.41 (2.86, 4,42)	3.30 (1.92, 6.93)	3.63 (0.96, 10.79)	5.44 (3.67, 7.07)	0.1925
**TNF-α$^+$/IL-17A$^+$ % T_H**	52.11 (43.05, 88.57)	104.00 (62.37, 143.90)	90.53 (25.22, 483.30)	86.28 (44.91, 230.00)	0.1381
**TNF-α/IL-17A MFI on T_H**	0.95 (0.83, 1.60)	2.01 (0.48, 3.83)	0.99 (0.27, 3.26)	1.90 (1.11, 2.75)	**0.0478**
**IFN-γ$^+$ % NK**	16.80 (8.72. 25.50)	9.38 (4.35, 48.60)	31.20 (2.39, 65.20)	13.05 (2.57, 41.50)	0.8136
**IFN-γ MFI on NK**	5018 (3170, 7333)	7212 (3070, 16769)	4771 (1448, 9862)	5450 (2257, 8993)	0.7291
**TNF-α$^+$ % NK**	3.40 (2.27, 5.57)	12.55 (3.96, 38.20)	6.52 (5.07, 12.70)	6.15 (0.62, 20.40)	**0.0146**
**TNF-α MFI on NK**	1254 (1101, 1541)	1829 (1114, 3034)	1532 (858, 2832)	1789 (1067, 4159)	0.0838
**IL-10$^+$ % NK**	0.29 (0.19, 0.41)	0.65 (0.24, 2.45)	0.85 (0.00, 1.81)	0.67 (0.07, 3.26)	0.0707
**IL-10 MFI on NK**	394 (344, 488)	477 (406, 1106)	329 (0, 1725)	524 (409, 1227)	0.1161
**RPL**					
**IL-2$^+$/IL-10$^+$ % T_H**	15.62 (4.95, 27.63)	14.58 (2.97, 113.90)	35.15 (2.91, 78.86)	58.85 (26.28, 97.10)	0.5032
**IL-2/IL-10 MFI on T_H**	1.84 (1.09, 3.42)	1.29 (0.84, 1.58)	1.23 (0.69, 2.04)	1.93 (0.76, 2.91)	0.4720
**IFN-γ$^+$/IL-10$^+$ % T_H**	9.98 (5.39, 26.54)	18.66 (4.15, 45.85)	32.31 (3.42, 75.44)	26.67 (19.19, 47.26)	0.6017
**IFN-γ/IL-10 MFI on T_H**	0.99 (0.53, 2.12)	0.64 (0.52, 1.16)	0.84 (0.41, 1.38)	0.77 (0.16, 2.72)	0.5062
**TNF-α$^+$/IL-10$^+$ % T_H**	40.94 (23.38, 90.00)	57.09 (9.98, 261.20)	129.50 (21.77, 186.80)	99.87 (57.38, 130.80)	0.6784
**TNF-α/IL-10 MFI on T_H**	0.42 (0.19, 0.60)	0.31 (0.17, 0.46)	0.23 (0.12, 0.48)	0.26 (0.14, 0.75)	0.9128
**IL-17A$^+$/IL-10$^+$ % T_H**	0.99 (0.59, 1.78)	1.05 (0.35, 2.21)	1.52 (0.29, 3.84)	0.98 (0.87, 1.28)	0.8499
**IL-17A/IL-10 MFI on T_H**	0.21 (0.11, 0.38)	0.11 (0.09, 0.24)	0.14 (0.07, 0.33)	0.41 (0.12, 0.51)	0.2871
**IL-2$^+$/IL-17A$^+$ % T_H**	21.53 (3.47, 40.48)	17.01 (9.94, 41.73)	39.80 (10.52, 49.17)	54.72 (27.61, 97.10)	0.3797
**IL-2/IL-17A MFI on T_H**	10.71 (8.01, 14.26)	10.55 (8.98, 13.95)	9.14 (6.18, 11.31)	5.61 (3.43, 6.44)	0.1162
**IFN-γ$^+$/IL-17A$^+$ % T_H**	10.79 (8.56, 14.90)	16.04 (9.77, 29.52)	15.12 (7.05, 21.43)	24.17 (20.72, 47.26)	0.1891
**IFN-γ/IL-17A MFI on T_H**	5.60 (3.58, 9.20)	4.68 (3.16, 7.47)	4.27 (3.47, 5.46)	2.77 (0.51, 5.33)	0.2363
**TNF-α$^+$/IL-17A$^+$ % T_H**	44.44 (28.08, 85.26)	55.73 (30.47, 95.22)	46.17 (18.34, 98.16)	95.73 (60.29, 130.80)	0.4429
**TNF-α/IL-17A MFI on T_H**	1.77 (1.23, 2.63)	1.57 (1.14, 3.17)	2.69 (1.15, 3.99)	1.05 (0.27, 2.69)	0.4206
**IFN-γ$^+$ % NK**	7.31 (2.06, 22.70)	8.12 (3.27, 20.00)	16.00 (5.37, 24.40)	38.20 (13.00, 58.10)	0.1694
**IFN-γ MFI on NK**	3137 (2249, 5533)	3151 (2665, 3761)	3676 (2987, 5336)	4316 (2025, 5652)	0.4252
**TNF-α$^+$ % NK**	1.82 (0.42, 3.41)	0.97 (0.41, 3.13)	0.97 (0.32, 7.41)	2.52 (0.08, 6.40)	0.8907
**TNF-α MFI on NK**	2211 (1481, 2882)	3045 (1498, 3855)	3380 (2132, 4556)	2929 (1579, 5737)	0.1954
**IL-10$^+$ % NK**	0.06 (0.00, 0.39)	0.01 (0.00, 0.17)	0.15 (0.00, 1.15)	0.00 (0.00, 0.03)	0.1195
**IL-10 MFI on NK**	412 (0, 497)	115 (0, 527)	555 (0, 844)	0 (0, 2148)	0.2595

***Supplementary table 7. Cytokine production in control women and women with recurrent pregnancy loss (RPL), belonging in different ethnic groups**. T_H cell ratios and NK cell cytokine production upon stimulation, assessed via the number of cytokine-producing cells and the per cell production represented via the medial fluorescence intensity (MFI), upon stimulation are displayed. For these non-normally distributed datasets, Kruskal-Wallis test was performed for control subgroup evaluation and Mann-Whitney test was used for comparisons within the RPL sub-cohort, with median and 95% confidence interval included.*

Parameter	Asian	Black	White	Mixed	p value
**Controls**					
**IL-2$^+$/IL-10$^+$ % T_H**	2.89 (0.14, 13.02)	1.10 (0.26, 57.90)	3.13 (1.48, 4.66)	13.00 (0.33, 405.20)	0.4233
**IL-2/IL-10 MFI on T_H**	6.91 (3.80, 9.61)	9.72 (0.89, 26.35)	6.88 (4.22, 8.73)	0.99 (0.45, 11.56)	0.0824
**IFN-γ$^+$/IL-10$^+$ % T_H**	1.05 (0.19, 9.16)	1.18 (0.23, 10.95)	1.26 (0.73, 2.88)	26.27 (0.18, 59.46)	0.3186
**IFN-γ/IL-10 MFI on T_H**	4.23 (0.99, 10.22)	1.11 (0.32, 2.33)	2.14 (1.19, 2.99)	0.41 (0.23, 1.96)	**0.0095**
**TNF-α$^+$/IL-10$^+$ % T_H**	4.32 (1.30, 20.71)	3.38 (1.11, 67.90)	7.58 (4.07, 11.30)	101.20 (0.94, 705.30)	0.3876
**TNF-α/IL-10 MFI on T_H**	0.60 (0.48, 1.15)	0.81 (0.09, 1.57)	0.46 (0.42, 0.58)	0.11 (0.04, 0.47)	**0.0061**
**IL-17A$^+$/IL-10$^+$ % T_H**	0.09 (0.01, 0.16)	0.05 (0.02, 1.31)	0.08 (0.04, 0.15)	3.05 (0.03, 16.38)	0.4110
**IL-17A/IL-10 MFI on T_H**	0.75 (0.33, 1.58)	0.44 (0.11, 1.40)	0.41 (0.37, 0.48)	0.16 (0.02, 0.53)	0.0544
**IL-2$^+$/IL-17A$^+$ % T_H**	23.69 (11.70, 103.90)	29.40 (8.05, 102.40)	25.35 (13.37, 37.13)	17.31 (0.71, 483.70)	0.9823
**IL-2/IL-17A MFI on T_H**	11.34 (3.49, 21.65)	16.40 (4.00, 51.66)	16.80 (9.39, 19.65)	15.32 (1.64, 41.32)	0.7125
**IFN-γ$^+$/IL-17A$^+$ % T_H**	16.87 (6.69, 73.04)	8.60 (5.53, 75.20)	14.34 (10.19, 17.89)	7.78 (2.98, 52.56)	0.6588
**IFN-γ/IL-17A MFI on T_H**	4.23 (1.61, 25.32)	2.21 (1.59, 3.74)	4.37 (3.28, 5.93)	4.72 (1.79, 9.40)	**0.0307**
**TNF-α$^+$/IL-17A$^+$ % T_H**	74.96 (30.92, 165.20)	53.87 (38.92, 229.60)	80.00 (59.21, 107.00)	38.05 (13.60, 341.90)	0.5433
**TNF-α/IL-17A MFI on T_H**	1.25 (0.39, 1.47)	1.90 (0.31, 3.26)	1.23 (0.89, 1.75)	1.43 (0.40, 1.70)	0.7912
**IFN-γ$^+$ % NK**	16.00 (8.33, 26.10)	38.90 (11.80, 65.20)	12.45 (7.63, 19.10)	4.66 (0.13, 57.30)	0.0818
**IFN-γ MFI on NK**	5018 (2257, 10248)	3010 (1448, 6502)	6288 (4771, 8411)	6777 (1421, 11746)	0.1231
**TNF-α$^+$ % NK**	10.90 (0.62, 42.90)	8.29 (3.04, 13.40)	5.25 (3.38, 6.74)	2.65 (0.34, 9.99)	0.2009
**TNF-α MFI on NK**	1392 (1232, 3107)	1537 (858, 3016)	1382 (1179, 1758)	1731 (633, 4352)	0.7574
**IL-10$^+$ % NK**	0.82 (0.08, 9.74)	0.72 (0.00, 1.81)	0.35 (0.27, 0.69)	0.09 (0.00, 1.04)	0.2818
**IL-10 MFI on NK**	431 (376, 980)	542 (0, 1725)	434 (378, 504)	481 (0. 2877)	0.8017
**RPL**					
**IL-2$^+$/IL-10$^+$ % T_H**	22.69 (2.03, 97.10)	-	6.64 (3.86, 20.96)	-	0.2977
**IL-2/IL-10 MFI on T_H**	0.94 (0.69, 3.42)	-	1.48 (1.23, 2.20)	-	0.3398
**IFN-γ$^+$/IL-10$^+$ % T_H**	41.24 (5.09, 85.24)	-	7.66 (4.37, 19.05)	-	0.0867
**IFN-γ/IL-10 MFI on T_H**	0.67 (0.33, 1.61)	-	0.88 (0.49, 1.17)	-	0.7365
**TNF-α$^+$/IL-10$^+$ % T_H**	131.30 (28.56, 268.40)	-	24.78 (14.83, 59.18)	-	0.0693
**TNF-α/IL-10 MFI on T_H**	0.17 (0.11, 0.45)	-	0.41 (0.24, 0.46)	-	0.0530
**IL-17A$^+$/IL-10$^+$ % T_H**	1.89 (0.29, 7.77)	-	0.77 (0.39, 1.39)	-	0.0789
**IL-17A/IL-10 MFI on T_H**	0.11 (0.06, 0.35)	-	0.20 (0.11, 0.31)	-	0.3253
**IL-2$^+$/IL-17A$^+$ % T_H**	24.92 (7.40, 42.45)	-	21.00 (9.94, 37.29)	-	0.9888
**IL-2/IL-17A MFI on T_H**	11.29 (8.98, 13.95)	-	10.31 (7.82, 11.70)	-	0.4480
**IFN-γ$^+$/IL-17A$^+$ % T_H**	12.20 (7.05, 18.28)	-	11.94 (8.68, 16.40)	-	0.7898
**IFN-γ/IL-17A MFI on T_H**	4.54 (3.47, 9.69)	-	4.36 (3.56, 5.46)	-	0.5001
**TNF-α$^+$/IL-17A$^+$ % T_H**	57.96 (29.88, 98.16)	-	44.00 (30.47, 63.03)	-	0.4737
**TNF-α/IL-17A MFI on T_H**	1.44 (1.12, 2.70)	-	1.67 (1.28, 2.78)	-	0.4230
**IFN-γ$^+$ % NK**	7.79 (2.81, 27.00)	-	8.17 (4.81, 19.60)	-	0.9180
**IFN-γ MFI on NK**	3813 (2871, 6173)	-	3026 (2655, 3469)	-	0.1081
**TNF-α$^+$ % NK**	0.96 (0.28, 3.41)	-	1.78 (0.75, 3.42)	-	0.5638
**TNF-α MFI on NK**	1901 (1152, 4159)	-	2369 (1860, 3253)	-	0.7422
**IL-10$^+$ % NK**	0.03 (0.00, 0.15)	-	0.21 (0.02, 0.39)	-	0.1193
**IL-10 MFI on NK**	258 (0, 908)	-	443 (286, 558)	-	0.6807
